# Hypertension Treatment in Nigeria (HTN) Program: rationale and design for a type 2 hybrid, effectiveness, and implementation interrupted time series trial

**DOI:** 10.1186/s43058-022-00328-9

**Published:** 2022-08-02

**Authors:** Abigail S. Baldridge, Kasarachi Aluka-Omitiran, Ikechukwu A. Orji, Gabriel L. Shedul, Tunde M. Ojo, Helen Eze, Grace Shedul, Eugenia N. Ugwuneji, Nonye B. Egenti, Rosemary C. B. Okoli, Boni M. Ale, Ada Nwankwo, Samuel Osagie, Jiancheng Ye, Aashima Chopra, Olutobi A. Sanuade, Priya Tripathi, Namratha R. Kandula, Lisa R. Hirschhorn, Mark D. Huffman, Dike B. Ojji

**Affiliations:** 1 Northwestern University Feinberg School of Medicine, Chicago, IL USA; 2 Cardiovascular Research Unit, University of Abuja Teaching Hospital, Abuja, Nigeria; 3 Federal Ministry of Health, Abuja, Nigeria; 4 University of Abuja, Abuja, Nigeria; 5 University of Nigeria, Nsukka, Nigeria; 6 Holo Healthcare, Nairobi, Kenya; 7 Spencer Fox Eccles School of Medicine, University of Utah, UT Salt Lake City, USA; 8 Stanley Manne Children’s Research Institute, Ann and Robert H. Lurie Children’s Hospital of Chicago, Chicago, IL USA; 9 Cardiovascular Division and Global Health Center, Washington University in St. Louis, St. Louis, MO USA; 10 The George Institute for Global Health, University of New South Wales, Sydney, Australia

**Keywords:** Implementation research, Hypertension, Task-shifting, Interrupted time series, Nigeria

## Abstract

**Background:**

Hypertension is the most common cardiovascular disease in Nigeria and contributes to a large non-communicable disease burden. Our aim was to implement and evaluate a large-scale hypertension treatment and control program, adapted from the Kaiser Permanent Northern California and World Health Organization HEARTS models, within public primary healthcare centers in the Federal Capital Territory, Nigeria.

**Methods:**

A type 2 hybrid, interrupted time series design was used to generate novel information on large-scale implementation and effectiveness of a multi-level hypertension control program within 60 primary healthcare centers in the Federal Capital Territory, Nigeria. During the formative phase, baseline qualitative assessments were held with patients, health workers, and administrators to inform implementation package adaptation. The package includes a hypertension patient registry with empanelment, performance and quality reporting, simplified treatment guideline emphasizing fixed-dose combination therapy, reliable access to quality essential medicines and technology, team-based care, and health coaching and home blood pressure monitoring. Strategies to implement and adapt the package were identified based on barriers and facilitators mapped in the formative phase, previous implementation experience, mid-term qualitative evaluation, and ongoing stakeholder and site feedback. The control phase included 11 months of sequential registration of hypertensive patients at participating primary healthcare centers, followed by implementation of the remainder of the package components and evaluation over 37 subsequent, consecutive months of the intervention phase.

The formative phase was completed between April 2019 and August 2019, followed by initiation of the control phase in January 2020. The control phase included 11 months (January 2020 to November 2020) of sequential registration and empanelment of hypertensive patients at participating primary healthcare centers. After completion of the control phase in November 2020, the intervention phase commenced in December 2020 and will be completed in December 2023.

**Discussion:**

This trial will provide robust evidence for implementation and effectiveness of a multi-level implementation package more broadly throughout the Federal Capital Territory, which may inform hypertension systems of care throughout Nigeria and in other low- and middle-income countries. Implementation outcome results will be important to understand what system-, site-, personnel-, and patient-level factors are necessary for successful implementation of this intervention.

**Trial registration:**

ClinicalTrials.gov NCT04158154. The trial was prospectively registered on November 8, 2019.

**Supplementary Information:**

The online version contains supplementary material available at 10.1186/s43058-022-00328-9.

Contributions to the literature

- Multi-level strategies for hypertension diagnosis, treatment, and control have rarely been implemented in Sub-Saharan Africa primary health care settings.
- Implementation of a contextually adapted hypertension treatment and control program within public primary health centers in the Federal Capital Territory, Nigeria, was informed by robust formative evaluation.
- The Hypertension Treatment in Nigeria Program is the largest primary-care based hypertension control program in Africa.
- Evaluation of implementation and effectiveness through a type 2 hybrid, interrupted time series design will provide robust evidence for scaling more broadly throughout the Federal Capital Territory, and potentially throughout Nigeria and in other low- and middle-income countries.

## Background

**Fig. 1:**
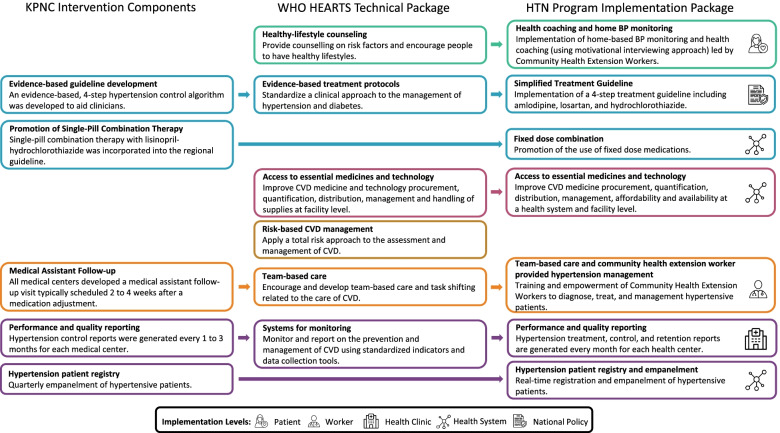
Alignment of the KPNC and WHO HEARTS technical packages with the Hypertension Treatment in Nigeria Program Implementation Package. The HTN Program implementation package is developed from the Kaiser Permanente Northern California and WHO HEARTS technical packages. Each component of the HTN Program implementation package is developed and deployed at different levels of the health system in the Federal Capital Territory, Nigeria. These levels include the patient, health worker, health clinic, health system, and national policy. All images are from Flaticon.com

In response to the global burden of cardiovascular disease, the World Health Organization (WHO) developed the HEARTS technical package, which is a strategy aimed at assisting ministries of health to manage cardiovascular diseases with a focus on primary health care settings in resource-limited contexts [1]. The WHO HEARTS technical package includes six modules which are informed by the Kaiser Permanente Northern California (KPNC) hypertension control program (Fig. 1) [2]. Several large-scale hypertension programs have adopted or adapted the HEARTS or KPNC models for implementation at the national and sub-national levels [3–6]. Though intended for use within resource-limited settings, implementation of the WHO HEARTS model within low- and middle-income countries requires systematic formative work to evaluate and address barriers to implementation, as evidenced by efforts of the Pan American Health Organization to introduce the model within 12 countries in the Americas [7].

Hypertension is the most common cardiovascular disease (CVD) in Nigeria, found in 86.4% of CVD patients, and is prevalent in an estimated 29–38% adult Nigerians [8, 9]. Multiple efforts are underway to address the burden of hypertension in Nigeria by increasing awareness, treatment, and control rates. For example, the National Hypertension Control Initiative (NHCI) is being piloted in 12 facilities in Ogun and Kano states to increase hypertension awareness, diagnosis, treatment, and control [10, 11]. The Hypertension Treatment in Nigeria (HTN) Program described in this report represents separate, yet related work. Both programs aim to lower hypertension-related morbidity and mortality, as well as strengthen hypertension diagnosis and management at the primary healthcare level through team-based care. More robust primary healthcare services will alleviate time and resource burdens on both patients and healthcare providers at secondary and tertiary care centers where hypertensive services have historically been provided in Nigeria.

Our aim was to implement and evaluate a large-scale hypertension treatment and control program within 60 public primary healthcare centers in the Federal Capital Territory, Nigeria through a type 2 hybrid effectiveness-implementation, interrupted time series design with outcomes measured using the RE-AIM framework [12].

## Methods/design

The methods described are based on the Standardized Reporting Items: Recommendations for Intervention Trials (SPIRIT) statement and checklist (Additional file 1) [13]. Details of the intervention and implementation strategies are described based on the Standards for Reporting Implementation Studies (StaRI) statement and checklist (Additional file 2) [14].

### Study design

The type 2 hybrid design was selected to generate novel information on large-scale implementation and effectiveness of a multi-level hypertension control program adapted from the KPNC intervention and WHO HEARTS technical package within primary healthcare centers in the Federal Capital Territory of Nigeria, and to inform subsequent plans for regional and national scale-up (Fig. 1). The interrupted time series design was selected as a pragmatic trial design which allows for the incorporation of contextual factors and underlying trends in treatment and control of hypertension [15]. Our study design allows for evaluation of health system, community, and individual-level characteristics that may affect the adaptation, effectiveness, and implementation of the HTN Program implementation package. The interrupted time series evaluation is accompanied by formative, interim, and follow-up qualitative assessments to simultaneously evaluate effectiveness and implementation. We chose not to use a randomized trial design given the high-quality evidence for our intervention components. A community health extension worker- (CHEW) led home blood pressure monitoring and health coaching sub-study was also included to evaluate implementation and effectiveness among randomly selected patients with persistently uncontrolled hypertension in 10 primary healthcare centers compared with propensity-matched controls. These individual-level strategies were selected because they have the largest effect sizes based on previous research [16].

### Timeline

**Fig. 2:**
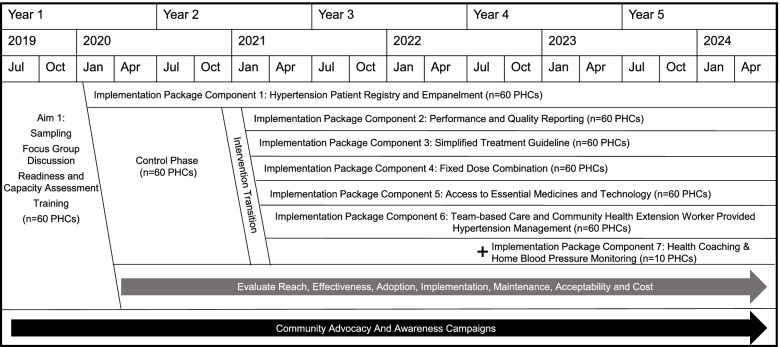
Timeline for the Hypertension Treatment in Nigeria Program. The HTN Program is implemented over 5 years, including a baseline formative phase, an 11-month control phase, and a 37-month intervention phase. Concurrent to the control and intervention phases, community advocacy and awareness campaigns are conducted within the Federal Capital Territory to increase awareness and demand for services.

During the formative phase of the study (April 2019 to August 2019), baseline qualitative assessments (i.e., key informant interviews and focus group discussions) were held with patients, health workers, and administrators to inform adaptation of the implementation package [17]. The control phase of the study included 11 months (January 2020 to November 2020) of sequential registration and empanelment of hypertensive patients at participating primary healthcare centers (Fig. 2). The intervention phase includes implementation of the remainder of the package components and evaluation over the subsequent 37 months (December 2020 to December 2023). Analysis and dissemination of baseline results occurred following conclusion of the control period [18]. Midway through the intervention phase, an additional component of CHEW-led home blood pressure monitoring and health coaching was introduced within a subset of 10 participating primary healthcare centers. Recruitment and follow-up of these patients will be carried out from September 2022 to February 2023.

### Participants

Prior to study initiation, we identified and assessed all public primary healthcare centers within the Federal Capital Territory of Nigeria (*N*=243) based on staffing, patient volumes, and functionality. To be eligible, primary healthcare centers had to have at least two full-time staff, be accessible to the study team by road and in all seasons, and be functional (i.e., defined as open at least between the hours of 8 AM and 4 PM and providing the minimum service package for primary healthcare centers). Sites were randomly selected through a multi-stage sampling frame including stratification by caseload and geography. Selected primary healthcare centers were invited to participate in the program, and all 60 (100%) of invited sites agreed to participate. Each selected primary healthcare center participated in a hypertension-adapted Service Availability and Readiness Assessment (SARA) based on the WHO instrument prior to initiation of the control phase to evaluate and confirm capacity and readiness for the study [19].

During the control and intervention phases, all adults (≥ 18 years) who presented at a participating site with persistently elevated blood pressure (systolic blood pressure ≥ 140 mmHg or diastolic blood pressure ≥90 mmHg, measured twice) or a history of hypertension were eligible for inclusion. Patients were registered in the study database through entry in a site-based patient registry log and empaneled in the hypertension cohort (Additional file 3). Informed consent was not collected per the Common Rule [20].

We collected data on paper case report forms, which were designed to be included in the patients’ medical files at each primary healthcare center (Additional file 4). Paper case report forms are completed and maintained by the CHEW at registration and each subsequent patient visit. After the patient visit, a record information officer at the site abstracted information from the patient case report form into the electronic REDCap database [21]. The REDCap database was hosted at the University of Abuja, and each participating primary healthcare center was provided with a tablet upon which the REDCap mobile application was installed. Data capture included, but was not limited to socio-demographics, medical history, anthropometrics, blood pressure values, relevant laboratory studies when available (e.g., serum potassium, serum creatinine), medications, side effects, adverse events, counseling, and patient vital status.

The CHEW-led home blood pressure monitoring and health coaching sub-study will focus on a subset of patients in the HTN Program. This strategy focuses on high-risk adults with uncontrolled hypertension despite participating in facility-based care among 10 selected primary healthcare centers. High-risk patients are defined as patients with elevated blood pressure (systolic blood pressure ≥ 140 mm Hg or diastolic blood pressure ≥ 90 mm Hg), who are 35 years and above and did not complete secondary school, the last characteristic serving as a marker of social disadvantage to prioritize this intervention among individuals with greater relative need for support.

### Intervention

The implementation package components were informed by the KPNC intervention toolkit and WHO HEARTS technical package (Fig. 1). To adapt the implementation package, we performed formative in-depth interviews and focus group discussions with patients, healthcare workers, and administrative staff to identify facilitators, barriers, and context to implementation of hypertension services within primary healthcare centers in the Federal Capital Territory, Nigeria [17]. The final implementation package includes a hypertension patient registry (health system level), performance and quality reporting (health clinic level), simplified treatment guidelines (national policy level), fixed-dose combination therapy (health system level), access to essential medicines and technology (health system level), team-based care and non-physician follow-up (health worker level), and health coaching and home blood pressure monitoring (patient level), all of which have demonstrated improved population-level hypertension control in other settings (Table 1) [3, 22, 23].

**Table 1:** Hypertension Treatment in Nigeria Program implementation package

Component	Start date	Number of sites	Level of intervention	Description as used in the HTN Program	Evaluation
Hypertension Patient Registry and Empanelment	January 2020	60	Health System	Paper-based registration and case report forms facilitate site-based tracking and follow-up of patients, aligns with existing practices for health record management, and are easy to implement. The electronic database facilitates rapid roll up of information in a structured database with built-in quality assurance checks.	Supportive supervision visits include comparison of the site visit logs and the hypertension registry to evaluate completeness of the registry. Bi-weekly data and safety quality reports are generated to review electronic data. Central and on-site training and reinforcement are provided, including the latter during site supervision visits to register all adult hypertensive patients.
Performance and Quality Reporting	December 2020	60	Health Clinic	Monthly performance and quality reports are issued to each participating site to summarize key metrics, aggregated over a rolling 3-month period. Metrics include treatment, control, and retention rates, along with lists of patients for targeted follow-up. Comparisons are made with other sites in the HTN Program.	Data quality audits are conducted during supportive supervision visits to compare data between the paper and electronic case report forms, and correct errant data points. Study team members communicate with sites on a quarterly basis to follow up on performance reports and ascertain if the site has reviewed and understood the report. In interim and final qualitative evaluations, sites will be asked for their feedback on the performance reports.
Simplified Treatment Guideline	December 2020	60	National Policy	A four-step simplified treatment guideline was enacted in the 2019 Nigeria Hypertension Protocol.	Medications data collected in the registry are used to calculate adherence to the four-step protocol by site, and across the program. In interim and final qualitative evaluations, sites will be asked for their feedback on the treatment guideline (appropriateness, feasibility, and adaptation).
Fixed-dose combination	December 2020	60	Health System	Fixed-dose combinations are encouraged in the hypertension treatment guideline when available.	Medications data collected in the registry are used to evaluate administration of fixed-dose or single pill prescriptions by site, and across the program. In interim and final qualitative evaluations, site will be asked for their feedback on fixed-dose combinations (appropriateness, feasibility, and adaptation).
Access to Essential Medicines and Technology	December 2020	60	Health System	Initial hypertension drug supplies were provided to all participating sites to prime the Drug Revolving Fund. During the first year of the intervention phase, work was completed to enact agreements for provision of hypertension medications at reasonable cost. The study team developed protocols, strategies, and training for adding hypertension medications to the Drug Revolving Fund.	Implementation of the Drug Revolving Fund will be evaluated in a separate protocol through RE-AIM outcomes. Early access to medicines in the study is evaluated through treatment rates, as well as stockout and replenishment rates at the participating sites.
Team-Based Care and Community Health Extension Worker Provided Hypertension Management	December 2020	60	Health Worker	Frontline workers (CHEWs, CHOs, pharmacists) have been trained during the control and intervention phase on components of the intervention package. A multidisciplinary team is involved in providing hypertension services in the participating health clinics.	Implementation of team-based care is evaluated through site supervision visits and provider reported experience.
Health Coaching and Home BP Monitoring	September 2022	10	Patient	CHEWs will lead a home-based blood pressure monitoring and health coaching (using motivational interviewing approach) intervention.	Implementation and effectiveness of the home blood pressure monitoring arm will be evaluated through a separate protocol including evaluation of blood pressures and pre- and post-intervention questionnaires.

*Abbreviations*: *CHEW* Community health extension worker, *CHO* Community health officer, *HTN* Hypertension Treatment in Nigeria

During the first 11 months (January 2020 through November 2020) of the program, all sites were in the control phase of the program during which time the only implementation package component used was the paper- and electronic-based patient registry. At initiation of the control phase, a 2-day, in-person training was provided to a minimum of two CHEWs from each participating site, which was focused on measurement of blood pressure, diagnosis of hypertension, and record management (Additional file 5) [24]. Pre- and post-workshop tests were conducted to evaluate changes in knowledge and practice, including evaluation of accurate blood pressure measurement. Record officers at each site were also trained through an in-person half-day training on electronic data management.

In December 2020, all sites transitioned to the intervention phase of the program. Healthcare workers participated in a full-day in-person training focused on a simplified treatment guideline following the Nigeria 2019 Hypertension Protocol, appropriate case management and patient treatment, and use of performance and quality reports [10]. The team trained six supervising Pharmacists/Pharmacy Technicians, one from each area council, and one pharmacy focal health worker from each of the 60 HTN primary healthcare centers on drug dispensing and logistics management before commencing the intervention phase. Primary healthcare center personnel were also retrained centrally at periodic intervals as well as quarterly, on-site supervision visits. Training was informed by feedback from audit and supervision reports. Moreover, all other full-time staff of the participating primary healthcare centers who were not among the healthcare workers initially selected for HTN Program were trained to build their capacity and to assist in the Program based on future needs. All training provided to staff at the participating primary healthcare centers was facilitated by supervisors who are members of the study team at University of Abuja Teaching Hospital.

In November 2021, a 2-day in-person training was provided to 30 CHEWs and 10 facility managers from 10 participating sites on how to carry out the home blood pressure and health coaching sub-study. The training focused on health coaching and motivational interviewing, screening consent at the participating sites, medication management, behavioral change related to hypertension, accurately measuring clinic and home blood pressure, health coaching visits, and role-playing. Pre-and post-training tests were conducted with CHEWs to understand changes in current knowledge and experience in health coaching and blood pressure monitoring. A second training session in April 2022 served as a refresher.

### Implementation strategies

Strategies to implement the package were identified based on barriers and facilitators mapping during the formative phase, previous implementation experience, mid-term qualitative evaluation, and ongoing stakeholder and site feedback (Table 2).

**Table 2:** Hypertension Treatment in Nigeria Program contextual factors and implementation strategies

Strategy for Implementing HTN Program	Level of implementation	Description	Implementation package component supported
*Use evaluative and iterative strategies*			
Assess for readiness and identify barriers and facilitators	Health facility	Formative work included quantitative evaluation of site-level readiness and capacity and qualitative evaluation of barriers and facilitators to implementation.	• Hypertension patient registry and empanelment • Team-based care and community health extension worker provided hypertension management
Audit and provide feedback	Health facility	Performance and quality reports are provided to each participating site on a monthly basis. Supportive supervision visits are performed quarterly (minimal semi-annually).	• Performance and quality reporting
Conduct local needs assessment	Program	A service availability and readiness assessment was performed during the formative phase of the HTN Program alongside qualitative evaluation of stakeholder (healthcare workers, supervisors, and patients) needs. For the duration of the program, stakeholders have been engaged through an advisory board, including a patient representative.	• Hypertension patient registry and empanelment • Team-based care and community health extension worker provided hypertension management • Access to essential medicines and technology
Develop and implement tools for quality monitoring	Health facility and Program	Simplified performance and quality reports were adapted from the WHO HEARTS reporting tools to focus on improving quality of care based on key indicators of hypertension treatment, control, and patient retention.	• Hypertension patient registry and empanelment • Performance and quality reporting • Fixed-dose combination
Involve patients/consumers and family members	Program	Patients were engaged in focus group discussions during the formative phase and during the interim and end-of-study assessments. A local patient representative sits on the advisory committee.	• Hypertension patient registry and empanelment
*Provide interactive assistance*			
Provide local technical assistance	Program	Technical assistance is provided to sites for data entry and correction by the study team coordinators at University of Abuja Teaching Hospital.	• Hypertension patient registry and empanelment • Performance and quality reporting
Provide clinical supervision	Program	Local area council physicians conduct clinical consultations within PHCs in their catchment areas. They may be called upon by CHEWs to discuss specific hypertensive patient cases. CHEWs may also call the research unit at UATH directly for patient case consultation and direct referral.	• Performance and quality reporting • Team-based care and community health extension worker provided hypertension management
*Adapt and tailor context*			
Tailor strategies	Health system	Strategies and implementation package component were locally adapted based on formative work. Emergent issues have driven adaptation to strategies and implementation package components, which are discussed by the operations team and enacted in a systematic way. A local context tracker is utilized to document emergent issues.	• Performance and quality reporting • Team-based care and community health extension worker provided hypertension management • Fixed-dose combination
*Develop stakeholder interrelationships*			
Inform local opinion leaders	Council Area and State	National and local area council public health leaders were included in the proposed program during the formative phase.	• Team-based care and community health extension worker provided hypertension management
Use advisory boards and workgroups	Program	An advisory committee was formed and convenes on an annual basis to inform and review program progress and evaluation.	• Team-based care and community health extension worker provided hypertension management • Access to essential medicines and technology
*Train and educate stakeholders*			
Conduct ongoing training	Program	Training is routinely provided to participating health care workers on components of the intervention and retraining as needed to reinforce quality data collection and adherence to the protocol.	• Performance and quality reporting • Simplified treatment guideline • Team-based care and community health extension worker provided hypertension management
Develop educational materials	Program	Contextually appropriate patient handouts and instructional materials were developed by the study team. Handouts depict the importance of health diets, regular physical exercise, smoking cessation, minimizing alcohol intake, weight loss, medication adherence and regular blood pressure checks. Community awareness campaigns are conducted in each area council to increase awareness of and demand for hypertension services.	• Simplified treatment guideline • Team-based care and community health extension worker provided hypertension management
Distribute educational materials	Health facility	Patient handouts are distributed by health educators during community awareness programs and by CHEWs during blood pressure screening visits within the PHCs.	• Hypertension patient registry and empanelment
Make training dynamic	Program	Demonstration-based learning techniques are used to reinforce information and methods for hypertension diagnosis, treatment, and management.	• Team-based care and community health extension worker provided hypertension management
Provide ongoing consultation	Health facility	Supportive supervision visits are conducted at least semi-annually to each participating health facility. Initial and ongoing training is provided to participating healthcare centers and CHEWs on the implementation components.	• Performance and quality reporting • Simplified treatment guideline • Team-based care and community health extension worker provided hypertension management
*Support clinicians*			
Create new clinical teams	Health facility	Team-based care (CHEWs, CHOs, Physicians, Medical Record Officers, Pharmacy Technicians, etc.) was provided at participating PHCs, focused on infectious diseases and maternal care. New teams specifically focused on hypertension care were formed or adapted for the HTN Program.	• Team-based care and community health extension worker provided hypertension management
Revise professional roles	Program	Encourage implementation of team-based care and task sharing.	• Team-based care and community health extension worker provided hypertension management
*Engage consumers*			
Increase demand	Health system	Conduct community outreach and mobilization activities to increase awareness and demand for hypertension services.	• Hypertension patient registry and Empanelment • Community awareness and mobilization campaigns
Intervene with patients/consumers to enhance uptake & adherence	Health system	Community awareness campaigns are conducted in each area council to increase awareness of and demand for hypertension services.	• Hypertension patient registry and empanelment • Community awareness and mobilization campaigns • Health coaching and home BP monitoring
*Utilize financial strategies*			
Alter incentive/allowance structures	Health worker	Frontline healthcare staff are compensated for registration of patients through monthly stipends of at least 10,000 naira each.	• Team-based care and community health extension worker provided hypertension management
Alter patient/consumer fees	Health system	Free or low-cost medicines are made available to hypertensive patients registered in the Program.	• Simplified treatment guideline • Fixed-dose combination • Access to essential medicines and technology
*Change infrastructure*			
Change physical structure and equipment	Health facility	All sites were equipped with functional automated blood pressure monitors, paper case report forms, electronic tablet, and data connections.	• Hypertension patient registry and empanelment
Change record system	Program	Create an electronic-based data capture system to supplement the paper-based system for rapid data collection and quality assurance.	• Performance and quality reporting
Change service sites	Program	Patients who would typically seek care in a tertiary care center are able to find the same hypertension care in their local health clinic. Home-based blood pressure monitoring and health coaching for individuals with persistently elevated blood pressure and social disadvantage	• Team-based care and community health extension worker provided hypertension management • Health coaching and home BP monitoring
Drug Revolving Fund	Health System	Addition of hypertension medications to the existing drug revolving fund.	• Simplified treatment guideline • Fixed-dose combination • Access to essential medicines and technology

*Abbreviations*: *BP* Blood pressure, *CHEW* Community health extension worker, *CHO* Community health officer, *HTN* Hypertension Treatment in Nigeria, *PHC* Primary healthcare center, *WHO* World Health Organization

### Community awareness campaign

A community awareness campaign was prospectively planned to accompany both baseline and intervention phases of the HTN Program. The purpose of the community awareness campaigns was to address misconceptions about hypertension, increase awareness at the community level, and to generate uptake of treatment of hypertension at the primary healthcare level. The campaign is led by members of the study team and implemented by health educators who are part of the primary healthcare workforce and local residents of the communities in which the campaigns are held. Within each area council, campaigns are held and reviewed quarterly.

### Outcomes

#### Primary outcomes

The study’s co-primary implementation outcomes include reach, effectiveness, adoption, implementation, maintenance, acceptability, and cost of the multi-level hypertension control program. The study's co-primary effectiveness outcomes are the change in slope from baseline slope of monthly hypertension treatment rates (defined as using any blood pressure-lowering drug) and monthly hypertension control rates (defined as systolic blood pressure <140 mmHg and diastolic blood pressure <90 mm Hg) among participating primary healthcare centers. We chose this definition of control based on the 2019 Nigeria Hypertension Protocol.

#### Secondary effectiveness

The study’s secondary effectiveness outcomes include (1) mean systolic blood pressure and diastolic blood pressure among eligible clinic patients with hypertension, and (2) rates of single versus two- or three-drug blood pressure-lowering medication use (including use of fixed-dose combination therapies).

#### Safety

The study’s safety outcomes include (1) proportion of participants with any potentially relevant side effect, including provider diagnosis of angioedema, acute kidney injury (defined as relative increase in serum creatinine by 50% or an absolute increase by 0.3 mg/dl [>0.26 μmol/L]), electrolyte abnormalities (defined as serum potassium < 3.5 or >5.5 mEq/L or serum sodium <125 or > 145 mEq/L), syncope, or dizziness, (2) rate of relevant side effects at the participant level (i.e., count per participant), and (3) proportion of participants with any serious adverse event according to Good Clinical Practice definition.

#### Implementation

Implementation outcomes focus on domains of the RE-AIM framework including at the program, center, and individual level (Table 3).

**Table 3:** Implementation outcomes for Hypertension Treatment in Nigeria Program

RE-AIM Domain: Definition	Level	Type	Outcome
Reach: Absolute number, proportion, and representativeness of sites and individuals who participate in the HTN Program	Program	Quantitative	• Number of participating PHCs/total number of selected PHCs in the Federal Capital Territory
	Center	Quantitative	• Diversity of participating PHCs and staff in terms of size, ward, baseline staffing levels
		Qualitative	• Reasons for non-participation of selected PHCs in the Federal Capital Territory • Reasons for adult patients to have not been screened for high BP within participating PHCs within the past 3 working days
	Individual	Quantitative	• Number of adult patients with BPs measured / total number of adult patients within participating PHCs within the past 3 working days • Differences in sociodemographic (e.g., age, sex, geography) characteristics between registered patients and individuals in the clinic catchment areas based on concurrently collected or community-based survey data • Diversity of registered patients receiving care at participating PHCs for HTN diagnosis and management by age, sex, ward, and education
Effectiveness: The impact of the HTN Program on treatment and control rates	Program	Quantitative	• Treatment rate within the overall system of participating PHCs defined by 6-month rolling average • Control rate within the overall system of participating PHCs defined by 6-month rolling average • Mean systolic blood pressure and diastolic blood pressure within the overall system of participating PHCs defined by 6-month rolling average and based on last visit
	Center	Quantitative	• Median and/or mean treatment rate across participating PHCs defined by 6-month rolling average • Median and/or mean control rate across participating PHCs defined by 6-month rolling average • Mean SBP and DBP across participating PHCs defined by 6-month rolling average and based on last visit
		Qualitative	• Reasons for variation in treatment rates between participating PHCs • Reasons for variation in control rates between participating PHCs • Reasons for variation in mean systolic and diastolic blood pressure between participating PHCs
Adoption: Absolute number, proportion, and representativeness of sites who are willing to initiate the HTN Program	Program	Quantitative	• Percentage of PHCs using the hypertension patient registry in the last 3 months • Percentage of patients treated with fixed-dose combination therapies in the last 3 months
		Qualitative	• Reasons for variation in registry use among participating PHCs at 3 months after site initiation • Reasons for variation in use of fixed-dose combination therapies in the last 3 months • Adoption of team-based care among participating PHCs, and reasons for success or challenges
Implementation: Fidelity to the HTN Program protocol, including consistency of delivery as intended. Time and cost of the intervention, and use of the intervention strategies	Program	Quantitative	Fidelity (Implementation) • Proportion of selected PHCs who participated in baseline hypertension training • Proportion of selected PHCs who participated in site initiation training • Proportion of selected PHCs who received at least one supportive supervision visit in the past 7 months • Proportion of selected PHCs who received an audit and feedback report within the past 3 months • Percentage of PHCs with a working blood pressure monitor at the site on the day of assessment • Percentage of PHCs with blood pressure medicines available on the day of assessment • Percentage of patients with step up indicated who received step up treatment in the last 6 months Cost • Modeled direct HTN Program costs based on staff, BP machines, data capture, data analysis, and BP lowering drugs for hypertension diagnosis, treatment and control overall, for each PHC and per patient
	Program	Qualitative	Fidelity (Implementation) • Reasons for variation in fidelity measures • Reasons for variation in availability of essentials medicines and equipment • Reasons for variation in fidelity to the step up treatment protocol Cost • Acceptability of upfront and ongoing HTN Program costs among stakeholders, including within Federal Ministry of Health
	Center	Quantitative	Fidelity (Intervention) • Number and proportion of adult patients with hypertension who are registered/total number of adult patients with elevated blood pressure within participating PHCs within the past 3 working days • Monthly proportion of registered patients with appropriate stepped care/total number of registered patients • Monthly proportion of registered patients treated with fixed-dose combination therapy/total number of patients on treatment
	Center, Individual	Qualitative	Fidelity (Implementation) • Reasons for adult patients with hypertension to have not been registered within participating PHCs within the past 3 working days
	Individual	Quantitative	Cost • Modeled monthly and annual out-of-pocket drug costs for hypertension treatment
	Individual	Qualitative	Acceptability • Reasons for variation in acceptability, satisfaction, and perceived quality of care at patient-level • Trust in primary health care system Cost • Acceptability of upfront and ongoing hypertension diagnosis and treatment costs among patients with hypertension
Maintenance: The extent to which the HTN Program becomes institutionalized or part of the routine organizational practice	Center	Quantitative	Maintenance • Proportion of participating PHCs who maintain treatment rates above baseline rates at 6, 12, 24, 36, and 48 months • Proportion of participating PHCs who maintain control rates above baseline rates at 6, 12, 24, 36, and 48 months • Proportion of participating PHCs without blood pressure medication stockouts at 36 and 48 months • Proportion of participants retained in care at participating PHCs at 6, 12, 24, 36, and 48 months
		Qualitative	Maintenance • Reasons for variation in maintenance of treatment rates above baseline rates • Reasons for variation in maintenance of control rates above baseline rates • Reasons for variation in sustainment of blood pressure medication supplies • Reasons for variation in proportion of participants retained in care at PHCs
	Individual	Qualitative	Maintenance • Reasons for remaining in care and on treatment within the PHC

*Abbreviations*: *BP* Blood pressure, *HTN* Hypertension Treatment in Nigeria, *PHC* Primary health care center

### Sample size

To estimate our sample size calculated for the primary effectiveness outcome, we used data from published literature to estimate the baseline prevalence of hypertension treatment (20%) and control (10%) rates in the Federal Capital Territory, Nigeria [25]. We based the anticipated effect on similar programs, including the KPNC Hypertension Control Program [2]. The power and sample size were estimated under a variety of conditions through Monte-Carlo simulation in SAS (v9.4, SAS Inc, Cary, North Carolina). Accounting for 10 sites dropping out, we estimated that 1200 patient visits per month across 50 sites over 9 months of baseline and 39 months of intervention would provide >80% power to detect a difference in treatment slope of 0.57% per month compared to underlying trend of 0.10% per month, resulting in hypertension treatment rate of 42.5% at the end of 48 months. Similarly, with the same sample size, we would have >80% power to detect a difference in control slope of 0.44% per month compared to underlying trend of 0.05% per month, resulting in hypertension control rate of 27.0% at the end of 48 months.

### Data quality assurance and control

To ensure data quality, we built in field validation(s) and branching logic within the REDCap forms. Before obtaining data entry rights, all study team members and healthcare workers with data access privileges completed training and pilot tested data entry using hypothetical data. Centralized statistical monitoring are performed through weekly data and status quality reports. The data and status quality reports use the REDCap application programming interface functionality to export the program data, and then are restructured and summarized using statistical software packages, including R (v4.0.3, R Core Team, Vienna, Austria) and SAS (v9.4, SAS Inc., Cary, NC). The reports are reviewed and discussed bi-weekly. Because the systems are in place and code has been generated, the reports can be updated in real time.

### Statistical analyses

**Fig 3:**
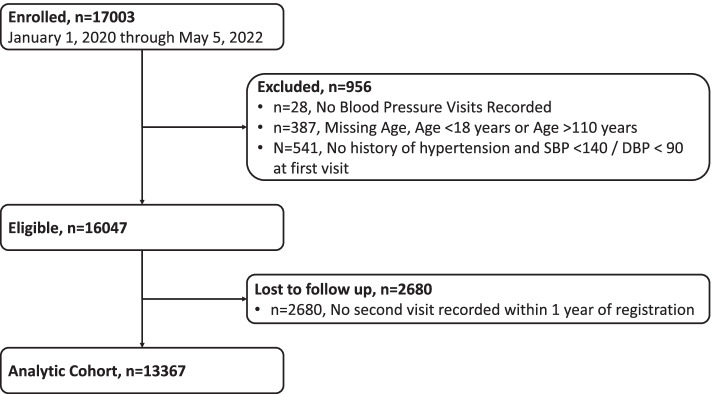
CONSORT Flowchart of Enrollment, Eligibility, and Analytic Sample Size in the Hypertension Treatment in Nigeria Program from January 1, 2020, through May 5, 2022

All participants who are enrolled in the patient registry will be accounted for, in accordance with the consolidated standards for reporting trials (CONSORT) statement for transparent reporting of trials (Fig. 3; Additional file 6). As of May 2022, more than 16,000 individuals participated in the Program. Baseline demographic variables, such as age, sex, and relevant clinical variables will be summarized when results are reported. Summaries of continuous baseline variables will be presented as means and standard deviations or medians with minimum and maximum values as appropriate and based on distribution. Categorical variables will be described as frequencies and percentages.

The statistical analysis will be performed using the intention to treat principle (i.e., all patient visits recorded in the database will be included and considered exposed to the intervention or unexposed according to the overall program timeline, regardless of when the intervention was actually implemented). The statistical design of the program intervention includes evaluation of effectiveness and implementation co-primary outcomes, which is consistent with the type 2 hybrid design. For implementation outcomes, we will use quantitative and qualitative analysis using the RE-AIM framework to triangulate routinely collected data to evaluate the reach, secondary effectiveness, adoption, implementation, maintenance, acceptability, and cost of the implementation package [12]. We will seek to explore and explain variability across key subgroups, including age, sex, and geography.

For effectiveness outcomes, we will use an interrupted time series evaluation with aggregate measures of system-level monthly treatment and control rates to evaluate temporal trends. We will evaluate change in temporal trends for primary and secondary outcomes through segmented linear regression by aggregating data across primary healthcare centers by month. To evaluate effects of each implementation package component and time variance in implementation on our primary outcomes, we will use patient-level mixed-effects models including random effects to account for clustering at the primary healthcare center level. We will perform sensitivity analyses by both excluding and restricting repeated measures of the same patients over the program period.

For the home blood pressure and health coaching sub-study, we will use analysis of covariance, controlling for baseline clinic-measured systolic blood pressure and medication to estimate the effect of a community health extension worker-led health coaching and home blood pressure monitoring on a 12-week change in systolic blood pressure compared with propensity-matched patients in the participating sites as controls. For implementation outcomes, we will adapt the RE-AIM framework and use quantitative data to evaluate the reach, effectiveness, adoption, implementation, maintenance, and cost of the interventions.

#### Subgroup analyses

Subgroup analyses will examine primary outcomes within and across sites based on staffing levels, staff training, geography, and drug availability. Exploratory subgroup analyses will similarly examine primary outcomes based on age, sex, duration of registration, and treatment regimen.

#### Interim analyses

No interim quantitative analyses for effectiveness are planned for this study. Interim qualitative analyses were performed in November 2021 to determine what adaptations should be made to the varied implementation strategies. Mid-term qualitative interviews were conducted in November 2021 to evaluate the implementation outcomes of the HTN Program using the RE-AIM framework. Seven focus group discussions were conducted with CHEWS or community health officers (*n*=3), facility managers (*n*=2), and primary care physicians (*n*=1). Rapid synthesis indicated that even though the HTN Program has been able to reach many patients, the Program reach is not equitable, with men and younger patients being less likely to participate than women and older patients [26, 27]. Additional community mobilization was suggested as a strategy to increase reach.

Regarding effectiveness, there was a general perception that the HTN Program strategies have been effective, especially with patient registration and empanelment, team-based care, and emphasis of fixed-dose combination therapy. Implementation of patient registration and empanelment, team-based care, and fixed-dose combination therapy has been feasible. However, achieving a high rate of monthly follow-up visits have been more challenging not only because of inherent challenges in retention and seasonal transportation issues, but also CHEW’s workload and security threats in some communities.

Regarding maintenance, there were serious concerns about the transition from freely available drugs to a Drug Revolving Fund-based system that emphasizes access through reduced out-of-pocket costs. Some participants mentioned that this may affect enrollment and retention.

#### Missing data

Missing data are identified through bi-weekly data quality reports and resolved with the responsible site where feasible. Complete case analyses will be performed in the primary analysis, and multiple imputation will be used for sensitivity analyses to explore the robustness of results.

### Monitoring

We have performed central statistical monitoring to evaluate sites’ performance and to direct risk-based, in-person, site monitoring and supportive supervision visits. In-person site monitoring visits have been conducted at minimum every 6 months with a target for quarterly visits for most sites. In-person visits are performed by at least two members of the HTN Program study team. Supportive supervision visits follow a checklist (Additional file 7) including quantification of site staff, inventory of equipment, observation of blood pressure measurement, and audit of patient paper case report forms. All site monitoring data are entered within a central REDCap database and summarized on a monthly basis to identify data quality problems, urgent staff or equipment needs, or other major data status or quality trends.

### Context tracker

A context tracker was developed for simultaneous capture of individual-, site-, regional-, national-, and global-level events that may affect implementation and effectiveness of the trial. The context tracker is maintained by two members of the study team and discussed monthly during operations team meetings. Contextual factors will be considered in sensitivity analyses to triangulate routinely collected data to evaluate the reach, secondary effectiveness, adoption, implementation, maintenance, acceptability, and cost of the implementation package.

## Discussion

The HTN Program aims to evaluate the effectiveness and implementation of hypertension services within primary healthcare centers in the Federal Capital Territory, Nigeria. The type 2 hybrid, interrupted time series design was employed because of several important features. First, simultaneous evaluation of implementation and effectiveness will generate information to inform adaptation and scaling of the intervention. Second, the interrupted time series design provides evidence for causal inference in evaluating the potential effects of an intervention compared with a pre-/post-intervention study design. Third, the design allows for evaluation of temporal trends, adaptation of implementation strategies, and assessment of the effects of contextual factors. Fourth, the interrupted time series design allows all primary healthcare centers to receive the intervention, which is important when the intervention is deemed likely to be beneficial. This design helps to avoid potential ethical concerns about sites being randomized to the control arm of a trial throughout the entire study period while having logistical advantages over a stepped wedge trial design given the large number of sites involved and perceived risk of site dropout, which was not realized.

However, the type 2 hybrid interrupted time series design also includes important limitations. We assumed a linear slope change effect between the control and intervention phases; however, this assumption may not be met in practice, limiting our ability to draw conclusions about the effectiveness of the intervention. The primary analysis of the HTN Program will employ segmented linear regression of aggregated measures of treatment and control at the system level, which does not account for imprecision at an individual patient or site level.

If successful in achieving its co-primary effectiveness outcomes, then this trial will provide robust evidence for implementation and effectiveness of this multi-level implementation package more broadly throughout the Federal Capital Territory, which may inform hypertension systems of care throughout Nigeria and potentially in other low- and middle-income countries. Data from our implementation outcomes will be important to understand what system-, site-, and personnel-level factors are necessary for successful implementation of this intervention.

## Supplementary Information

**Additional file 1.** SPIRIT Checklist. Completed SPIRIT checklist for the HTN Program protocol manuscript.

**Additional file 2.** StaRI Checklist. Completed StaRI checklist for the HTN Program protocol manuscript.

**Additional file 3.** HTN Program Registry Log. Template of the site-based HTN Program registry logbook.

**Additional file 4.** HTN Program Case Report Form. Template of the site-based paper case report form for the HTN Program which is included in each patients’ medical records.

**Additional file 5.** HTN Program Training. Detailed listing of training provided to HTN Program community health extension workers, community health officers, record officers, pharmacists, and other healthcare workers.

**Additional file 6.** HTN Program Statistical Analysis Plan. Statistical analysis plan for the HTN Program.

**Additional file 7.** HTN Program Supervision Checklist. Checklist used by members of the HTN Program implementation team during usual supervision visits to participating sites.

## Data Availability

The datasets generated and/or analyzed during the current study will be available in the NHLBI BioLINCC repository, https://biolincc.nhlbi.nih.gov/home/ after completion of the study.

## Abbreviations

CHEW: Community health extension worker
CONSORT: Consolidated Standards for Reporting Trials
CVD: Cardiovascular disease
DSQR: Data Status Quality Report
HTN: Hypertension Treatment in Nigeria
NHCI: National Hypertension Control Initiative
KPNC: Kaiser Permanent Northern California
REDCap: Research Electronic Data Capture
SPIRIT: Standardized Reporting Items: Recommendations for Intervention Trials
StaRI: Standards for Reporting Implementation Studies
WHO: World Health Organization
