# Supplementary material for: Hypertension Treatment in Nigeria (HTN) Program: rationale and design for a type 2 hybrid, effectiveness, and implementation interrupted time series trial
Source: Implement Sci Commun. 2022 Aug 2;3:84. doi: 10.1186/s43058-022-00328-9 (PMC9344662; doi:10.1186/s43058-022-00328-9)
Supplement: Supplementary file 3 — Additional file 3. HTN Program Registry Log. Template of the site-based HTN Program registry logbook. [file 43058_2022_328_MOESM3_ESM.pdf]

HTN Enrollment Registry: Site Number

V1.2 27 January 2020

Facility Information

Council Area:	Ward:	Health Center Name:

Patient Identification Information

Patient ID#	Registration Date (DD/MM/YY)	New HTN Diagnosis	Unique ID	Surname	First Name	Gender	Date of Birth (DD/MM/YYYY)	Phone Number 1	Next of Kin Name	Next of Kin Phone Number
0001	__ __/__ __/__ __	☐Y ☐ N				☐M ☐ F	__ __/__ __/__ __ __ __			
0002	__ __/__ __/__ __	☐Y ☐ N				☐M ☐ F	__ __/__ __/__ __ __ __			
0003	__ __/__ __/__ __	☐Y ☐ N				☐M ☐ F	__ __/__ __/__ __ __ __			
0004	__ __/__ __/__ __	☐Y ☐ N				☐M ☐ F	__ __/__ __/__ __ __ __			
0005	__ __/__ __/__ __	☐Y ☐ N				☐M ☐ F	__ __/__ __/__ __ __ __			
0006	__ __/__ __/__ __	☐Y ☐ N				☐M ☐ F	__ __/__ __/__ __ __ __			
0007	__ __/__ __/__ __	☐Y ☐ N				☐M ☐ F	__ __/__ __/__ __ __ __			
0008	__ __/__ __/__ __	☐Y ☐ N				☐M ☐ F	__ __/__ __/__ __ __ __			
0009	__ __/__ __/__ __	☐Y ☐ N				☐M ☐ F	__ __/__ __/__ __ __ __			
0010	__ __/__ __/__ __	☐Y ☐ N				☐M ☐ F	__ __/__ __/__ __ __ __			
0011	__ __/__ __/__ __	☐Y ☐ N				☐M ☐ F	__ __/__ __/__ __ __ __			
0012	__ __/__ __/__ __	☐Y ☐ N				☐M ☐ F	__ __/__ __/__ __ __ __			
0013	__ __/__ __/__ __	☐Y ☐ N				☐M ☐ F	__ __/__ __/__ __ __ __			
0014	__ __/__ __/__ __	☐Y ☐ N				☐M ☐ F	__ __/__ __/__ __ __ __			
0015	__ __/__ __/__ __	☐Y ☐ N				☐M ☐ F	__ __/__ __/__ __ __ __			
0016	__ __/__ __/__ __	☐Y ☐ N				☐M ☐ F	__ __/__ __/__ __ __ __			
0017	__ __/__ __/__ __	☐Y ☐ N				☐M ☐ F	__ __/__ __/__ __ __ __			
0018	__ __/__ __/__ __	☐Y ☐ N				☐M ☐ F	__ __/__ __/__ __ __ __			
0019	__ __/__ __/__ __	☐Y ☐ N				☐M ☐ F	__ __/__ __/__ __ __ __			
0020	__ __/__ __/__ __	☐Y ☐ N				☐M ☐ F	__ __/__ __/__ __ __ __			
0021	__ __/__ __/__ __	☐Y ☐ N				☐M ☐ F	__ __/__ __/__ __ __ __			
0022	__ __/__ __/__ __	☐Y ☐ N				☐M ☐ F	__ __/__ __/__ __ __ __			
0023	__ __/__ __/__ __	☐Y ☐ N				☐M ☐ F	__ __/__ __/__ __ __ __			
0024	__ __/__ __/__ __	☐Y ☐ N				☐M ☐ F	__ __/__ __/__ __ __ __			
0025	__ __/__ __/__ __	☐Y ☐ N				☐M ☐ F	__ __/__ __/__ __ __ __			
0026	__ __/__ __/__ __	☐Y ☐ N				☐M ☐ F	__ __/__ __/__ __ __ __			
0027	__ __/__ __/__ __	☐Y ☐ N				☐M ☐ F	__ __/__ __/__ __ __ __			
0028	__ __/__ __/__ __	☐Y ☐ N				☐M ☐ F	__ __/__ __/__ __ __ __			
0029	__ __/__ __/__ __	☐Y ☐ N				☐M ☐ F	__ __/__ __/__ __ __ __			
0030	__ __/__ __/__ __	☐Y ☐ N				☐M ☐ F	__ __/__ __/__ __ __ __			
0031	__ __/__ __/__ __	☐Y ☐ N				☐M ☐ F	__ __/__ __/__ __ __ __			
0032	__ __/__ __/__ __	☐Y ☐ N				☐M ☐ F	__ __/__ __/__ __ __ __			
0033	__ __/__ __/__ __	☐Y ☐ N				☐M ☐ F	__ __/__ __/__ __ __ __			
0034	__ __/__ __/__ __	☐Y ☐ N				☐M ☐ F	__ __/__ __/__ __ __ __			

HTN Enrollment Registry: Site Number

V1.2 27 January 2020

Patient Identification Information

Patient ID#	Registration Date (DD/MM/YY)	New HTN Diagnosis	Unique ID	Surname	First Name	Gender	Date of Birth (DD/MM/YYYY)	Phone Number 1	Next of Kin Name	Next of Kin Phone Number
0035	__ __/__ __/__ __	☐Y ☐ N				☐M ☐ F	__ __/__ __/__ __ __ __			
0036	__ __/__ __/__ __	☐Y ☐ N				☐M ☐ F	__ __/__ __/__ __ __ __			
0037	__ __/__ __/__ __	☐Y ☐ N				☐M ☐ F	__ __/__ __/__ __ __ __			
0038	__ __/__ __/__ __	☐Y ☐ N				☐M ☐ F	__ __/__ __/__ __ __ __			
0039	__ __/__ __/__ __	☐Y ☐ N				☐M ☐ F	__ __/__ __/__ __ __ __			
0040	__ __/__ __/__ __	☐Y ☐ N				☐M ☐ F	__ __/__ __/__ __ __ __			
0041	__ __/__ __/__ __	☐Y ☐ N				☐M ☐ F	__ __/__ __/__ __ __ __			
0042	__ __/__ __/__ __	☐Y ☐ N				☐M ☐ F	__ __/__ __/__ __ __ __			
0043	__ __/__ __/__ __	☐Y ☐ N				☐M ☐ F	__ __/__ __/__ __ __ __			
0044	__ __/__ __/__ __	☐Y ☐ N				☐M ☐ F	__ __/__ __/__ __ __ __			
0045	__ __/__ __/__ __	☐Y ☐ N				☐M ☐ F	__ __/__ __/__ __ __ __			
0046	__ __/__ __/__ __	☐Y ☐ N				☐M ☐ F	__ __/__ __/__ __ __ __			
0047	__ __/__ __/__ __	☐Y ☐ N				☐M ☐ F	__ __/__ __/__ __ __ __			
0048	__ __/__ __/__ __	☐Y ☐ N				☐M ☐ F	__ __/__ __/__ __ __ __			
0049	__ __/__ __/__ __	☐Y ☐ N				☐M ☐ F	__ __/__ __/__ __ __ __			
0050	__ __/__ __/__ __	☐Y ☐ N				☐M ☐ F	__ __/__ __/__ __ __ __			
0051	__ __/__ __/__ __	☐Y ☐ N				☐M ☐ F	__ __/__ __/__ __ __ __			
0052	__ __/__ __/__ __	☐Y ☐ N				☐M ☐ F	__ __/__ __/__ __ __ __			
0053	__ __/__ __/__ __	☐Y ☐ N				☐M ☐ F	__ __/__ __/__ __ __ __			
0054	__ __/__ __/__ __	☐Y ☐ N				☐M ☐ F	__ __/__ __/__ __ __ __			
0055	__ __/__ __/__ __	☐Y ☐ N				☐M ☐ F	__ __/__ __/__ __ __ __			
0056	__ __/__ __/__ __	☐Y ☐ N				☐M ☐ F	__ __/__ __/__ __ __ __			
0057	__ __/__ __/__ __	☐Y ☐ N				☐M ☐ F	__ __/__ __/__ __ __ __			
0058	__ __/__ __/__ __	☐Y ☐ N				☐M ☐ F	__ __/__ __/__ __ __ __			
0059	__ __/__ __/__ __	☐Y ☐ N				☐M ☐ F	__ __/__ __/__ __ __ __			
0060	__ __/__ __/__ __	☐Y ☐ N				☐M ☐ F	__ __/__ __/__ __ __ __			
0061	__ __/__ __/__ __	☐Y ☐ N				☐M ☐ F	__ __/__ __/__ __ __ __			
0062	__ __/__ __/__ __	☐Y ☐ N				☐M ☐ F	__ __/__ __/__ __ __ __			
0063	__ __/__ __/__ __	☐Y ☐ N				☐M ☐ F	__ __/__ __/__ __ __ __			
0064	__ __/__ __/__ __	☐Y ☐ N				☐M ☐ F	__ __/__ __/__ __ __ __			
0065	__ __/__ __/__ __	☐Y ☐ N				☐M ☐ F	__ __/__ __/__ __ __ __			
0066	__ __/__ __/__ __	☐Y ☐ N				☐M ☐ F	__ __/__ __/__ __ __ __			
0067	__ __/__ __/__ __	☐Y ☐ N				☐M ☐ F	__ __/__ __/__ __ __ __			
0068	__ __/__ __/__ __	☐Y ☐ N				☐M ☐ F	__ __/__ __/__ __ __ __			
0069	__ __/__ __/__ __	☐Y ☐ N				☐M ☐ F	__ __/__ __/__ __ __ __			
0070	__ __/__ __/__ __	☐Y ☐ N				☐M ☐ F	__ __/__ __/__ __ __ __			

HTN Enrollment Registry: Site Number

V1.2 27 January 2020

Patient Identification Information

Patient ID#	Registration Date (DD/MM/YY)	New HTN Diagnosis	Unique ID	Surname	First Name	Gender	Date of Birth (DD/MM/YYYY)	Phone Number 1	Next of Kin Name	Next of Kin Phone Number
0071	__ __/__ __/__ __	☐Y ☐ N				☐M ☐ F	__ __/__ __/__ __ __ __			
0072	__ __/__ __/__ __	☐Y ☐ N				☐M ☐ F	__ __/__ __/__ __ __ __			
0073	__ __/__ __/__ __	☐Y ☐ N				☐M ☐ F	__ __/__ __/__ __ __ __			
0074	__ __/__ __/__ __	☐Y ☐ N				☐M ☐ F	__ __/__ __/__ __ __ __			
0075	__ __/__ __/__ __	☐Y ☐ N				☐M ☐ F	__ __/__ __/__ __ __ __			
0076	__ __/__ __/__ __	☐Y ☐ N				☐M ☐ F	__ __/__ __/__ __ __ __			
0077	__ __/__ __/__ __	☐Y ☐ N				☐M ☐ F	__ __/__ __/__ __ __ __			
0078	__ __/__ __/__ __	☐Y ☐ N				☐M ☐ F	__ __/__ __/__ __ __ __			
0079	__ __/__ __/__ __	☐Y ☐ N				☐M ☐ F	__ __/__ __/__ __ __ __			
0080	__ __/__ __/__ __	☐Y ☐ N				☐M ☐ F	__ __/__ __/__ __ __ __			
0081	__ __/__ __/__ __	☐Y ☐ N				☐M ☐ F	__ __/__ __/__ __ __ __			
0082	__ __/__ __/__ __	☐Y ☐ N				☐M ☐ F	__ __/__ __/__ __ __ __			
0083	__ __/__ __/__ __	☐Y ☐ N				☐M ☐ F	__ __/__ __/__ __ __ __			
0084	__ __/__ __/__ __	☐Y ☐ N				☐M ☐ F	__ __/__ __/__ __ __ __			
0085	__ __/__ __/__ __	☐Y ☐ N				☐M ☐ F	__ __/__ __/__ __ __ __			
0086	__ __/__ __/__ __	☐Y ☐ N				☐M ☐ F	__ __/__ __/__ __ __ __			
0087	__ __/__ __/__ __	☐Y ☐ N				☐M ☐ F	__ __/__ __/__ __ __ __			
0088	__ __/__ __/__ __	☐Y ☐ N				☐M ☐ F	__ __/__ __/__ __ __ __			
0089	__ __/__ __/__ __	☐Y ☐ N				☐M ☐ F	__ __/__ __/__ __ __ __			
0090	__ __/__ __/__ __	☐Y ☐ N				☐M ☐ F	__ __/__ __/__ __ __ __			
0091	__ __/__ __/__ __	☐Y ☐ N				☐M ☐ F	__ __/__ __/__ __ __ __			
0092	__ __/__ __/__ __	☐Y ☐ N				☐M ☐ F	__ __/__ __/__ __ __ __			
0093	__ __/__ __/__ __	☐Y ☐ N				☐M ☐ F	__ __/__ __/__ __ __ __			
0094	__ __/__ __/__ __	☐Y ☐ N				☐M ☐ F	__ __/__ __/__ __ __ __			
0095	__ __/__ __/__ __	☐Y ☐ N				☐M ☐ F	__ __/__ __/__ __ __ __			
0096	__ __/__ __/__ __	☐Y ☐ N				☐M ☐ F	__ __/__ __/__ __ __ __			
0097	__ __/__ __/__ __	☐Y ☐ N				☐M ☐ F	__ __/__ __/__ __ __ __			
0098	__ __/__ __/__ __	☐Y ☐ N				☐M ☐ F	__ __/__ __/__ __ __ __			
0099	__ __/__ __/__ __	☐Y ☐ N				☐M ☐ F	__ __/__ __/__ __ __ __			
0100	__ __/__ __/__ __	☐Y ☐ N				☐M ☐ F	__ __/__ __/__ __ __ __			
0101	__ __/__ __/__ __	☐Y ☐ N				☐M ☐ F	__ __/__ __/__ __ __ __			
0102	__ __/__ __/__ __	☐Y ☐ N				☐M ☐ F	__ __/__ __/__ __ __ __			
0103	__ __/__ __/__ __	☐Y ☐ N				☐M ☐ F	__ __/__ __/__ __ __ __			
0104	__ __/__ __/__ __	☐Y ☐ N				☐M ☐ F	__ __/__ __/__ __ __ __			
0105	__ __/__ __/__ __	☐Y ☐ N				☐M ☐ F	__ __/__ __/__ __ __ __			
0106	__ __/__ __/__ __	☐Y ☐ N				☐M ☐ F	__ __/__ __/__ __ __ __			

HTN Enrollment Registry: Site Number

V1.2 27 January 2020

Patient Identification Information

Patient ID#	Registration Date (DD/MM/YY)	New HTN Diagnosis	Unique ID	Surname	First Name	Gender	Date of Birth (DD/MM/YYYY)	Phone Number 1	Next of Kin Name	Next of Kin Phone Number
0107	__ __/__ __/__ __	☐Y ☐ N				☐M ☐ F	__ __/__ __/__ __ __ __			
0108	__ __/__ __/__ __	☐Y ☐ N				☐M ☐ F	__ __/__ __/__ __ __ __			
0109	__ __/__ __/__ __	☐Y ☐ N				☐M ☐ F	__ __/__ __/__ __ __ __			
0110	__ __/__ __/__ __	☐Y ☐ N				☐M ☐ F	__ __/__ __/__ __ __ __			
0111	__ __/__ __/__ __	☐Y ☐ N				☐M ☐ F	__ __/__ __/__ __ __ __			
0112	__ __/__ __/__ __	☐Y ☐ N				☐M ☐ F	__ __/__ __/__ __ __ __			
0113	__ __/__ __/__ __	☐Y ☐ N				☐M ☐ F	__ __/__ __/__ __ __ __			
0114	__ __/__ __/__ __	☐Y ☐ N				☐M ☐ F	__ __/__ __/__ __ __ __			
0115	__ __/__ __/__ __	☐Y ☐ N				☐M ☐ F	__ __/__ __/__ __ __ __			
0116	__ __/__ __/__ __	☐Y ☐ N				☐M ☐ F	__ __/__ __/__ __ __ __			
0117	__ __/__ __/__ __	☐Y ☐ N				☐M ☐ F	__ __/__ __/__ __ __ __			
0118	__ __/__ __/__ __	☐Y ☐ N				☐M ☐ F	__ __/__ __/__ __ __ __			
0119	__ __/__ __/__ __	☐Y ☐ N				☐M ☐ F	__ __/__ __/__ __ __ __			
0120	__ __/__ __/__ __	☐Y ☐ N				☐M ☐ F	__ __/__ __/__ __ __ __			
0121	__ __/__ __/__ __	☐Y ☐ N				☐M ☐ F	__ __/__ __/__ __ __ __			
0122	__ __/__ __/__ __	☐Y ☐ N				☐M ☐ F	__ __/__ __/__ __ __ __			
0123	__ __/__ __/__ __	☐Y ☐ N				☐M ☐ F	__ __/__ __/__ __ __ __			
0124	__ __/__ __/__ __	☐Y ☐ N				☐M ☐ F	__ __/__ __/__ __ __ __			
0125	__ __/__ __/__ __	☐Y ☐ N				☐M ☐ F	__ __/__ __/__ __ __ __			
0126	__ __/__ __/__ __	☐Y ☐ N				☐M ☐ F	__ __/__ __/__ __ __ __			
0127	__ __/__ __/__ __	☐Y ☐ N				☐M ☐ F	__ __/__ __/__ __ __ __			
0128	__ __/__ __/__ __	☐Y ☐ N				☐M ☐ F	__ __/__ __/__ __ __ __			
0129	__ __/__ __/__ __	☐Y ☐ N				☐M ☐ F	__ __/__ __/__ __ __ __			
0130	__ __/__ __/__ __	☐Y ☐ N				☐M ☐ F	__ __/__ __/__ __ __ __			
0131	__ __/__ __/__ __	☐Y ☐ N				☐M ☐ F	__ __/__ __/__ __ __ __			
0132	__ __/__ __/__ __	☐Y ☐ N				☐M ☐ F	__ __/__ __/__ __ __ __			
0133	__ __/__ __/__ __	☐Y ☐ N				☐M ☐ F	__ __/__ __/__ __ __ __			
0134	__ __/__ __/__ __	☐Y ☐ N				☐M ☐ F	__ __/__ __/__ __ __ __			
0135	__ __/__ __/__ __	☐Y ☐ N				☐M ☐ F	__ __/__ __/__ __ __ __			
0136	__ __/__ __/__ __	☐Y ☐ N				☐M ☐ F	__ __/__ __/__ __ __ __			
0137	__ __/__ __/__ __	☐Y ☐ N				☐M ☐ F	__ __/__ __/__ __ __ __			
0138	__ __/__ __/__ __	☐Y ☐ N				☐M ☐ F	__ __/__ __/__ __ __ __			
0139	__ __/__ __/__ __	☐Y ☐ N				☐M ☐ F	__ __/__ __/__ __ __ __			
0140	__ __/__ __/__ __	☐Y ☐ N				☐M ☐ F	__ __/__ __/__ __ __ __			
0141	__ __/__ __/__ __	☐Y ☐ N				☐M ☐ F	__ __/__ __/__ __ __ __			
0142	__ __/__ __/__ __	☐Y ☐ N				☐M ☐ F	__ __/__ __/__ __ __ __			

HTN Enrollment Registry: Site Number

V1.2 27 January 2020

Patient Identification Information

Patient ID#	Registration Date (DD/MM/YY)	New HTN Diagnosis	Unique ID	Surname	First Name	Gender	Date of Birth (DD/MM/YYYY)	Phone Number 1	Next of Kin Name	Next of Kin Phone Number
0143	__ __/__ __/__ __	☐Y ☐N				☐M ☐F	__ __/__ __/__ __ __ __			
0144	__ __/__ __/__ __	☐Y ☐N				☐M ☐F	__ __/__ __/__ __ __ __			
0145	__ __/__ __/__ __	☐Y ☐N				☐M ☐F	__ __/__ __/__ __ __ __			
0146	__ __/__ __/__ __	☐Y ☐N				☐M ☐F	__ __/__ __/__ __ __ __			
0147	__ __/__ __/__ __	☐Y ☐N				☐M ☐F	__ __/__ __/__ __ __ __			
0148	__ __/__ __/__ __	☐Y ☐N				☐M ☐F	__ __/__ __/__ __ __ __			
0149	__ __/__ __/__ __	☐Y ☐N				☐M ☐F	__ __/__ __/__ __ __ __			
0150	__ __/__ __/__ __	☐Y ☐N				☐M ☐F	__ __/__ __/__ __ __ __			
0151	__ __/__ __/__ __	☐Y ☐N				☐M ☐F	__ __/__ __/__ __ __ __			
0152	__ __/__ __/__ __	☐Y ☐N				☐M ☐F	__ __/__ __/__ __ __ __			
0153	__ __/__ __/__ __	☐Y ☐N				☐M ☐F	__ __/__ __/__ __ __ __			
0154	__ __/__ __/__ __	☐Y ☐N				☐M ☐F	__ __/__ __/__ __ __ __			
0155	__ __/__ __/__ __	☐Y ☐N				☐M ☐F	__ __/__ __/__ __ __ __			
0156	__ __/__ __/__ __	☐Y ☐N				☐M ☐F	__ __/__ __/__ __ __ __			
0157	__ __/__ __/__ __	☐Y ☐N				☐M ☐F	__ __/__ __/__ __ __ __			
0158	__ __/__ __/__ __	☐Y ☐N				☐M ☐F	__ __/__ __/__ __ __ __			
0159	__ __/__ __/__ __	☐Y ☐N				☐M ☐F	__ __/__ __/__ __ __ __			
0160	__ __/__ __/__ __	☐Y ☐N				☐M ☐F	__ __/__ __/__ __ __ __			
0161	__ __/__ __/__ __	☐Y ☐N				☐M ☐F	__ __/__ __/__ __ __ __			
0162	__ __/__ __/__ __	☐Y ☐N				☐M ☐F	__ __/__ __/__ __ __ __			
0163	__ __/__ __/__ __	☐Y ☐N				☐M ☐F	__ __/__ __/__ __ __ __			
0164	__ __/__ __/__ __	☐Y ☐N				☐M ☐F	__ __/__ __/__ __ __ __			
0165	__ __/__ __/__ __	☐Y ☐N				☐M ☐F	__ __/__ __/__ __ __ __			
0166	__ __/__ __/__ __	☐Y ☐N				☐M ☐F	__ __/__ __/__ __ __ __			
0167	__ __/__ __/__ __	☐Y ☐N				☐M ☐F	__ __/__ __/__ __ __ __			
0168	__ __/__ __/__ __	☐Y ☐N				☐M ☐F	__ __/__ __/__ __ __ __			
0169	__ __/__ __/__ __	☐Y ☐N				☐M ☐F	__ __/__ __/__ __ __ __			
0170	__ __/__ __/__ __	☐Y ☐N				☐M ☐F	__ __/__ __/__ __ __ __			
0171	__ __/__ __/__ __	☐Y ☐N				☐M ☐F	__ __/__ __/__ __ __ __			
0172	__ __/__ __/__ __	☐Y ☐N				☐M ☐F	__ __/__ __/__ __ __ __			
0173	__ __/__ __/__ __	☐Y ☐N				☐M ☐F	__ __/__ __/__ __ __ __			
0174	__ __/__ __/__ __	☐Y ☐N				☐M ☐F	__ __/__ __/__ __ __ __			
0175	__ __/__ __/__ __	☐Y ☐N				☐M ☐F	__ __/__ __/__ __ __ __			
0176	__ __/__ __/__ __	☐Y ☐N				☐M ☐F	__ __/__ __/__ __ __ __			
0177	__ __/__ __/__ __	☐Y ☐N				☐M ☐F	__ __/__ __/__ __ __ __			
0178	__ __/__ __/__ __	☐Y ☐N				☐M ☐F	__ __/__ __/__ __ __ __			

HTN Enrollment Registry: Site Number

V1.2 27 January 2020

Patient Identification Information

Patient ID#	Registration Date (DD/MM/YY)	New HTN Diagnosis	Unique ID	Surname	First Name	Gender	Date of Birth (DD/MM/YYYY)	Phone Number 1	Next of Kin Name	Next of Kin Phone Number
0179	__ __/__ __/__ __	☐Y ☐N				☐M ☐F	__ __/__ __/__ __ __ __			
0180	__ __/__ __/__ __	☐Y ☐N				☐M ☐F	__ __/__ __/__ __ __ __			
0181	__ __/__ __/__ __	☐Y ☐N				☐M ☐F	__ __/__ __/__ __ __ __			
0182	__ __/__ __/__ __	☐Y ☐N				☐M ☐F	__ __/__ __/__ __ __ __			
0183	__ __/__ __/__ __	☐Y ☐N				☐M ☐F	__ __/__ __/__ __ __ __			
0184	__ __/__ __/__ __	☐Y ☐N				☐M ☐F	__ __/__ __/__ __ __ __			
0185	__ __/__ __/__ __	☐Y ☐N				☐M ☐F	__ __/__ __/__ __ __ __			
0186	__ __/__ __/__ __	☐Y ☐N				☐M ☐F	__ __/__ __/__ __ __ __			
0187	__ __/__ __/__ __	☐Y ☐N				☐M ☐F	__ __/__ __/__ __ __ __			
0188	__ __/__ __/__ __	☐Y ☐N				☐M ☐F	__ __/__ __/__ __ __ __			
0189	__ __/__ __/__ __	☐Y ☐N				☐M ☐F	__ __/__ __/__ __ __ __			
0190	__ __/__ __/__ __	☐Y ☐N				☐M ☐F	__ __/__ __/__ __ __ __			
0191	__ __/__ __/__ __	☐Y ☐N				☐M ☐F	__ __/__ __/__ __ __ __			
0192	__ __/__ __/__ __	☐Y ☐N				☐M ☐F	__ __/__ __/__ __ __ __			
0193	__ __/__ __/__ __	☐Y ☐N				☐M ☐F	__ __/__ __/__ __ __ __			
0194	__ __/__ __/__ __	☐Y ☐N				☐M ☐F	__ __/__ __/__ __ __ __			
0195	__ __/__ __/__ __	☐Y ☐N				☐M ☐F	__ __/__ __/__ __ __ __			
0196	__ __/__ __/__ __	☐Y ☐N				☐M ☐F	__ __/__ __/__ __ __ __			
0197	__ __/__ __/__ __	☐Y ☐N				☐M ☐F	__ __/__ __/__ __ __ __			
0198	__ __/__ __/__ __	☐Y ☐N				☐M ☐F	__ __/__ __/__ __ __ __			
0199	__ __/__ __/__ __	☐Y ☐N				☐M ☐F	__ __/__ __/__ __ __ __			
0200	__ __/__ __/__ __	☐Y ☐N				☐M ☐F	__ __/__ __/__ __ __ __			
0201	__ __/__ __/__ __	☐Y ☐N				☐M ☐F	__ __/__ __/__ __ __ __			
0202	__ __/__ __/__ __	☐Y ☐N				☐M ☐F	__ __/__ __/__ __ __ __			
0203	__ __/__ __/__ __	☐Y ☐N				☐M ☐F	__ __/__ __/__ __ __ __			
0204	__ __/__ __/__ __	☐Y ☐N				☐M ☐F	__ __/__ __/__ __ __ __			
0205	__ __/__ __/__ __	☐Y ☐N				☐M ☐F	__ __/__ __/__ __ __ __			
0206	__ __/__ __/__ __	☐Y ☐N				☐M ☐F	__ __/__ __/__ __ __ __			
0207	__ __/__ __/__ __	☐Y ☐N				☐M ☐F	__ __/__ __/__ __ __ __			
0208	__ __/__ __/__ __	☐Y ☐N				☐M ☐F	__ __/__ __/__ __ __ __			
0209	__ __/__ __/__ __	☐Y ☐N				☐M ☐F	__ __/__ __/__ __ __ __			
0210	__ __/__ __/__ __	☐Y ☐N				☐M ☐F	__ __/__ __/__ __ __ __			
0211	__ __/__ __/__ __	☐Y ☐N				☐M ☐F	__ __/__ __/__ __ __ __			
0212	__ __/__ __/__ __	☐Y ☐N				☐M ☐F	__ __/__ __/__ __ __ __			
0213	__ __/__ __/__ __	☐Y ☐N				☐M ☐F	__ __/__ __/__ __ __ __			
0214	__ __/__ __/__ __	☐Y ☐N				☐M ☐F	__ __/__ __/__ __ __ __			

HTN Enrollment Registry: Site Number

V1.2 27 January 2020

Patient Identification Information

Patient ID#	Registration Date (DD/MM/YY)	New HTN Diagnosis	Unique ID	Surname	First Name	Gender	Date of Birth (DD/MM/YYYY)	Phone Number 1	Next of Kin Name	Next of Kin Phone Number
0215	__ __/__ __/__ __	☐Y ☐N				☐M ☐F	__ __/__ __/__ __ __ __			
0216	__ __/__ __/__ __	☐Y ☐N				☐M ☐F	__ __/__ __/__ __ __ __			
0217	__ __/__ __/__ __	☐Y ☐N				☐M ☐F	__ __/__ __/__ __ __ __			
0218	__ __/__ __/__ __	☐Y ☐N				☐M ☐F	__ __/__ __/__ __ __ __			
0219	__ __/__ __/__ __	☐Y ☐N				☐M ☐F	__ __/__ __/__ __ __ __			
0220	__ __/__ __/__ __	☐Y ☐N				☐M ☐F	__ __/__ __/__ __ __ __			
0221	__ __/__ __/__ __	☐Y ☐N				☐M ☐F	__ __/__ __/__ __ __ __			
0222	__ __/__ __/__ __	☐Y ☐N				☐M ☐F	__ __/__ __/__ __ __ __			
0223	__ __/__ __/__ __	☐Y ☐N				☐M ☐F	__ __/__ __/__ __ __ __			
0224	__ __/__ __/__ __	☐Y ☐N				☐M ☐F	__ __/__ __/__ __ __ __			
0225	__ __/__ __/__ __	☐Y ☐N				☐M ☐F	__ __/__ __/__ __ __ __			
0226	__ __/__ __/__ __	☐Y ☐N				☐M ☐F	__ __/__ __/__ __ __ __			
0227	__ __/__ __/__ __	☐Y ☐N				☐M ☐F	__ __/__ __/__ __ __ __			
0228	__ __/__ __/__ __	☐Y ☐N				☐M ☐F	__ __/__ __/__ __ __ __			
0229	__ __/__ __/__ __	☐Y ☐N				☐M ☐F	__ __/__ __/__ __ __ __			
0230	__ __/__ __/__ __	☐Y ☐N				☐M ☐F	__ __/__ __/__ __ __ __			
0231	__ __/__ __/__ __	☐Y ☐N				☐M ☐F	__ __/__ __/__ __ __ __			
0232	__ __/__ __/__ __	☐Y ☐N				☐M ☐F	__ __/__ __/__ __ __ __			
0233	__ __/__ __/__ __	☐Y ☐N				☐M ☐F	__ __/__ __/__ __ __ __			
0234	__ __/__ __/__ __	☐Y ☐N				☐M ☐F	__ __/__ __/__ __ __ __			
0235	__ __/__ __/__ __	☐Y ☐N				☐M ☐F	__ __/__ __/__ __ __ __			
0236	__ __/__ __/__ __	☐Y ☐N				☐M ☐F	__ __/__ __/__ __ __ __			
0237	__ __/__ __/__ __	☐Y ☐N				☐M ☐F	__ __/__ __/__ __ __ __			
0238	__ __/__ __/__ __	☐Y ☐N				☐M ☐F	__ __/__ __/__ __ __ __			
0239	__ __/__ __/__ __	☐Y ☐N				☐M ☐F	__ __/__ __/__ __ __ __			
0240	__ __/__ __/__ __	☐Y ☐N				☐M ☐F	__ __/__ __/__ __ __ __			
0241	__ __/__ __/__ __	☐Y ☐N				☐M ☐F	__ __/__ __/__ __ __ __			
0242	__ __/__ __/__ __	☐Y ☐N				☐M ☐F	__ __/__ __/__ __ __ __			
0243	__ __/__ __/__ __	☐Y ☐N				☐M ☐F	__ __/__ __/__ __ __ __			
0244	__ __/__ __/__ __	☐Y ☐N				☐M ☐F	__ __/__ __/__ __ __ __			
0245	__ __/__ __/__ __	☐Y ☐N				☐M ☐F	__ __/__ __/__ __ __ __			
0246	__ __/__ __/__ __	☐Y ☐N				☐M ☐F	__ __/__ __/__ __ __ __			
0247	__ __/__ __/__ __	☐Y ☐N				☐M ☐F	__ __/__ __/__ __ __ __			
0248	__ __/__ __/__ __	☐Y ☐N				☐M ☐F	__ __/__ __/__ __ __ __			
0249	__ __/__ __/__ __	☐Y ☐N				☐M ☐F	__ __/__ __/__ __ __ __			
0250	__ __/__ __/__ __	☐Y ☐N				☐M ☐F	__ __/__ __/__ __ __ __			

HTN Enrollment Registry: Site Number V1.2 27 January 2020

Patient Identification Information

Patient ID#	Registration Date (DD/MM/YY)	New HTN Diagnosis	Unique ID	Surname	First Name	Gender	Date of Birth (DD/MM/YYYY)	Phone Number 1	Next of Kin Name	Next of Kin Phone Number
0251	__ __/__ __/__ __	☐Y ☐ N				☐M ☐ F	__ __/__ __/__ __ __ __			
0252	__ __/__ __/__ __	☐Y ☐ N				☐M ☐ F	__ __/__ __/__ __ __ __			
0253	__ __/__ __/__ __	☐Y ☐ N				☐M ☐ F	__ __/__ __/__ __ __ __			
0254	__ __/__ __/__ __	☐Y ☐ N				☐M ☐ F	__ __/__ __/__ __ __ __			
0255	__ __/__ __/__ __	☐Y ☐ N				☐M ☐ F	__ __/__ __/__ __ __ __			
0256	__ __/__ __/__ __	☐Y ☐ N				☐M ☐ F	__ __/__ __/__ __ __ __			
0257	__ __/__ __/__ __	☐Y ☐ N				☐M ☐ F	__ __/__ __/__ __ __ __			
0258	__ __/__ __/__ __	☐Y ☐ N				☐M ☐ F	__ __/__ __/__ __ __ __			
0259	__ __/__ __/__ __	☐Y ☐ N				☐M ☐ F	__ __/__ __/__ __ __ __			
0260	__ __/__ __/__ __	☐Y ☐ N				☐M ☐ F	__ __/__ __/__ __ __ __			
0261	__ __/__ __/__ __	☐Y ☐ N				☐M ☐ F	__ __/__ __/__ __ __ __			
0262	__ __/__ __/__ __	☐Y ☐ N				☐M ☐ F	__ __/__ __/__ __ __ __			
0263	__ __/__ __/__ __	☐Y ☐ N				☐M ☐ F	__ __/__ __/__ __ __ __			
0264	__ __/__ __/__ __	☐Y ☐ N				☐M ☐ F	__ __/__ __/__ __ __ __			
0265	__ __/__ __/__ __	☐Y ☐ N				☐M ☐ F	__ __/__ __/__ __ __ __			
0266	__ __/__ __/__ __	☐Y ☐ N				☐M ☐ F	__ __/__ __/__ __ __ __			
0267	__ __/__ __/__ __	☐Y ☐ N				☐M ☐ F	__ __/__ __/__ __ __ __			
0268	__ __/__ __/__ __	☐Y ☐ N				☐M ☐ F	__ __/__ __/__ __ __ __			
0269	__ __/__ __/__ __	☐Y ☐ N				☐M ☐ F	__ __/__ __/__ __ __ __			
0270	__ __/__ __/__ __	☐Y ☐ N				☐M ☐ F	__ __/__ __/__ __ __ __			
0271	__ __/__ __/__ __	☐Y ☐ N				☐M ☐ F	__ __/__ __/__ __ __ __			
0272	__ __/__ __/__ __	☐Y ☐ N				☐M ☐ F	__ __/__ __/__ __ __ __			
0273	__ __/__ __/__ __	☐Y ☐ N				☐M ☐ F	__ __/__ __/__ __ __ __			
0274	__ __/__ __/__ __	☐Y ☐ N				☐M ☐ F	__ __/__ __/__ __ __ __			
0275	__ __/__ __/__ __	☐Y ☐ N				☐M ☐ F	__ __/__ __/__ __ __ __			
0276	__ __/__ __/__ __	☐Y ☐ N				☐M ☐ F	__ __/__ __/__ __ __ __			
0277	__ __/__ __/__ __	☐Y ☐ N				☐M ☐ F	__ __/__ __/__ __ __ __			
0278	__ __/__ __/__ __	☐Y ☐ N				☐M ☐ F	__ __/__ __/__ __ __ __			
0279	__ __/__ __/__ __	☐Y ☐ N				☐M ☐ F	__ __/__ __/__ __ __ __			
0280	__ __/__ __/__ __	☐Y ☐ N				☐M ☐ F	__ __/__ __/__ __ __ __			
0281	__ __/__ __/__ __	☐Y ☐ N				☐M ☐ F	__ __/__ __/__ __ __ __			
0282	__ __/__ __/__ __	☐Y ☐ N				☐M ☐ F	__ __/__ __/__ __ __ __			
0283	__ __/__ __/__ __	☐Y ☐ N				☐M ☐ F	__ __/__ __/__ __ __ __			
0284	__ __/__ __/__ __	☐Y ☐ N				☐M ☐ F	__ __/__ __/__ __ __ __			
0285	__ __/__ __/__ __	☐Y ☐ N				☐M ☐ F	__ __/__ __/__ __ __ __			
0286	__ __/__ __/__ __	☐Y ☐ N				☐M ☐ F	__ __/__ __/__ __ __ __			

HTN Enrollment Registry: Site Number

V1.2 27 January 2020

Patient Identification Information

Patient ID#	Registration Date (DD/MM/YY)	New HTN Diagnosis	Unique ID	Surname	First Name	Gender	Date of Birth (DD/MM/YYYY)	Phone Number 1	Next of Kin Name	Next of Kin Phone Number
0287	__ __/__ __/__ __	☐Y ☐N				☐M ☐F	__ __/__ __/__ __ __ __			
0288	__ __/__ __/__ __	☐Y ☐N				☐M ☐F	__ __/__ __/__ __ __ __			
0289	__ __/__ __/__ __	☐Y ☐N				☐M ☐F	__ __/__ __/__ __ __ __			
0290	__ __/__ __/__ __	☐Y ☐N				☐M ☐F	__ __/__ __/__ __ __ __			
0291	__ __/__ __/__ __	☐Y ☐N				☐M ☐F	__ __/__ __/__ __ __ __			
0292	__ __/__ __/__ __	☐Y ☐N				☐M ☐F	__ __/__ __/__ __ __ __			
0293	__ __/__ __/__ __	☐Y ☐N				☐M ☐F	__ __/__ __/__ __ __ __			
0294	__ __/__ __/__ __	☐Y ☐N				☐M ☐F	__ __/__ __/__ __ __ __			
0295	__ __/__ __/__ __	☐Y ☐N				☐M ☐F	__ __/__ __/__ __ __ __			
0296	__ __/__ __/__ __	☐Y ☐N				☐M ☐F	__ __/__ __/__ __ __ __			
0297	__ __/__ __/__ __	☐Y ☐N				☐M ☐F	__ __/__ __/__ __ __ __			
0298	__ __/__ __/__ __	☐Y ☐N				☐M ☐F	__ __/__ __/__ __ __ __			
0299	__ __/__ __/__ __	☐Y ☐N				☐M ☐F	__ __/__ __/__ __ __ __			
0300	__ __/__ __/__ __	☐Y ☐N				☐M ☐F	__ __/__ __/__ __ __ __			
0301	__ __/__ __/__ __	☐Y ☐N				☐M ☐F	__ __/__ __/__ __ __ __			
0302	__ __/__ __/__ __	☐Y ☐N				☐M ☐F	__ __/__ __/__ __ __ __			
0303	__ __/__ __/__ __	☐Y ☐N				☐M ☐F	__ __/__ __/__ __ __ __			
0304	__ __/__ __/__ __	☐Y ☐N				☐M ☐F	__ __/__ __/__ __ __ __			
0305	__ __/__ __/__ __	☐Y ☐N				☐M ☐F	__ __/__ __/__ __ __ __			
0306	__ __/__ __/__ __	☐Y ☐N				☐M ☐F	__ __/__ __/__ __ __ __			
0307	__ __/__ __/__ __	☐Y ☐N				☐M ☐F	__ __/__ __/__ __ __ __			
0308	__ __/__ __/__ __	☐Y ☐N				☐M ☐F	__ __/__ __/__ __ __ __			
0309	__ __/__ __/__ __	☐Y ☐N				☐M ☐F	__ __/__ __/__ __ __ __			
0310	__ __/__ __/__ __	☐Y ☐N				☐M ☐F	__ __/__ __/__ __ __ __			
0311	__ __/__ __/__ __	☐Y ☐N				☐M ☐F	__ __/__ __/__ __ __ __			
0312	__ __/__ __/__ __	☐Y ☐N				☐M ☐F	__ __/__ __/__ __ __ __			
0313	__ __/__ __/__ __	☐Y ☐N				☐M ☐F	__ __/__ __/__ __ __ __			
0314	__ __/__ __/__ __	☐Y ☐N				☐M ☐F	__ __/__ __/__ __ __ __			
0315	__ __/__ __/__ __	☐Y ☐N				☐M ☐F	__ __/__ __/__ __ __ __			
0316	__ __/__ __/__ __	☐Y ☐N				☐M ☐F	__ __/__ __/__ __ __ __			
0317	__ __/__ __/__ __	☐Y ☐N				☐M ☐F	__ __/__ __/__ __ __ __			
0318	__ __/__ __/__ __	☐Y ☐N				☐M ☐F	__ __/__ __/__ __ __ __			
0319	__ __/__ __/__ __	☐Y ☐N				☐M ☐F	__ __/__ __/__ __ __ __			
0320	__ __/__ __/__ __	☐Y ☐N				☐M ☐F	__ __/__ __/__ __ __ __			
0321	__ __/__ __/__ __	☐Y ☐N				☐M ☐F	__ __/__ __/__ __ __ __			
0322	__ __/__ __/__ __	☐Y ☐N				☐M ☐F	__ __/__ __/__ __ __ __			

HTN Enrollment Registry: Site Number

V1.2 27 January 2020

Patient Identification Information

Patient ID#	Registration Date (DD/MM/YY)	New HTN Diagnosis	Unique ID	Surname	First Name	Gender	Date of Birth (DD/MM/YYYY)	Phone Number 1	Next of Kin Name	Next of Kin Phone Number
0323	__ __/__ __/__ __	☐Y ☐ N				☐M ☐ F	__ __/__ __/__ __ __ __			
0324	__ __/__ __/__ __	☐Y ☐ N				☐M ☐ F	__ __/__ __/__ __ __ __			
0325	__ __/__ __/__ __	☐Y ☐ N				☐M ☐ F	__ __/__ __/__ __ __ __			
0326	__ __/__ __/__ __	☐Y ☐ N				☐M ☐ F	__ __/__ __/__ __ __ __			
0327	__ __/__ __/__ __	☐Y ☐ N				☐M ☐ F	__ __/__ __/__ __ __ __			
0328	__ __/__ __/__ __	☐Y ☐ N				☐M ☐ F	__ __/__ __/__ __ __ __			
0329	__ __/__ __/__ __	☐Y ☐ N				☐M ☐ F	__ __/__ __/__ __ __ __			
0330	__ __/__ __/__ __	☐Y ☐ N				☐M ☐ F	__ __/__ __/__ __ __ __			
0331	__ __/__ __/__ __	☐Y ☐ N				☐M ☐ F	__ __/__ __/__ __ __ __			
0332	__ __/__ __/__ __	☐Y ☐ N				☐M ☐ F	__ __/__ __/__ __ __ __			
0333	__ __/__ __/__ __	☐Y ☐ N				☐M ☐ F	__ __/__ __/__ __ __ __			
0334	__ __/__ __/__ __	☐Y ☐ N				☐M ☐ F	__ __/__ __/__ __ __ __			
0335	__ __/__ __/__ __	☐Y ☐ N				☐M ☐ F	__ __/__ __/__ __ __ __			
0336	__ __/__ __/__ __	☐Y ☐ N				☐M ☐ F	__ __/__ __/__ __ __ __			
0337	__ __/__ __/__ __	☐Y ☐ N				☐M ☐ F	__ __/__ __/__ __ __ __			
0338	__ __/__ __/__ __	☐Y ☐ N				☐M ☐ F	__ __/__ __/__ __ __ __			
0339	__ __/__ __/__ __	☐Y ☐ N				☐M ☐ F	__ __/__ __/__ __ __ __			
0340	__ __/__ __/__ __	☐Y ☐ N				☐M ☐ F	__ __/__ __/__ __ __ __			
0341	__ __/__ __/__ __	☐Y ☐ N				☐M ☐ F	__ __/__ __/__ __ __ __			
0342	__ __/__ __/__ __	☐Y ☐ N				☐M ☐ F	__ __/__ __/__ __ __ __			
0343	__ __/__ __/__ __	☐Y ☐ N				☐M ☐ F	__ __/__ __/__ __ __ __			
0344	__ __/__ __/__ __	☐Y ☐ N				☐M ☐ F	__ __/__ __/__ __ __ __			
0345	__ __/__ __/__ __	☐Y ☐ N				☐M ☐ F	__ __/__ __/__ __ __ __			
0346	__ __/__ __/__ __	☐Y ☐ N				☐M ☐ F	__ __/__ __/__ __ __ __			
0347	__ __/__ __/__ __	☐Y ☐ N				☐M ☐ F	__ __/__ __/__ __ __ __			
0348	__ __/__ __/__ __	☐Y ☐ N				☐M ☐ F	__ __/__ __/__ __ __ __			
0349	__ __/__ __/__ __	☐Y ☐ N				☐M ☐ F	__ __/__ __/__ __ __ __			
0350	__ __/__ __/__ __	☐Y ☐ N				☐M ☐ F	__ __/__ __/__ __ __ __			
0351	__ __/__ __/__ __	☐Y ☐ N				☐M ☐ F	__ __/__ __/__ __ __ __			
0352	__ __/__ __/__ __	☐Y ☐ N				☐M ☐ F	__ __/__ __/__ __ __ __			
0353	__ __/__ __/__ __	☐Y ☐ N				☐M ☐ F	__ __/__ __/__ __ __ __			
0354	__ __/__ __/__ __	☐Y ☐ N				☐M ☐ F	__ __/__ __/__ __ __ __			
0355	__ __/__ __/__ __	☐Y ☐ N				☐M ☐ F	__ __/__ __/__ __ __ __			
0356	__ __/__ __/__ __	☐Y ☐ N				☐M ☐ F	__ __/__ __/__ __ __ __			
0357	__ __/__ __/__ __	☐Y ☐ N				☐M ☐ F	__ __/__ __/__ __ __ __			
0358	__ __/__ __/__ __	☐Y ☐ N				☐M ☐ F	__ __/__ __/__ __ __ __			
