# Supplementary material for: Hypertension Treatment in Nigeria (HTN) Program: rationale and design for a type 2 hybrid, effectiveness, and implementation interrupted time series trial
Source: Implement Sci Commun. 2022 Aug 2;3:84. doi: 10.1186/s43058-022-00328-9 (PMC9344662; doi:10.1186/s43058-022-00328-9)
Supplement: Supplementary file 4 — Additional file 4. HTN Program Case Report Form. Template of the site-based paper case report form for the HTN Program which is included in each patients’ medical records. [file 43058_2022_328_MOESM4_ESM.pdf]

J. Side Effects, complete for each event reported

Date Event Started (DD/MM/YY)	Date Event Stopped (DD/MM/YY)	Effect Type: Cough	Angioedema	Acute kidney injury	Electrolyte abnormality	Syncope	Dizziness	Required medical treatment
___/___/___	___/___/___	☐	☐	☐	☐	☐	☐	☐Yes ☐No
___/___/___	___/___/___	☐	☐	☐	☐	☐	☐	☐Yes ☐No
___/___/___	___/___/___	☐	☐	☐	☐	☐	☐	☐Yes ☐No
___/___/___	___/___/___	☐	☐	☐	☐	☐	☐	☐Yes ☐No
___/___/___	___/___/___	☐	☐	☐	☐	☐	☐	☐Yes ☐No
___/___/___	___/___/___	☐	☐	☐	☐	☐	☐	☐Yes ☐No
___/___/___	___/___/___	☐	☐	☐	☐	☐	☐	☐Yes ☐No
___/___/___	___/___/___	☐	☐	☐	☐	☐	☐	☐Yes ☐No
___/___/___	___/___/___	☐	☐	☐	☐	☐	☐	☐Yes ☐No
___/___/___	___/___/___	☐	☐	☐	☐	☐	☐	☐Yes ☐No
___/___/___	___/___/___	☐	☐	☐	☐	☐	☐	☐Yes ☐No
___/___/___	___/___/___	☐	☐	☐	☐	☐	☐	☐Yes ☐No

K. Serious Adverse Events

Date Event Started (DD/MM/YY)	Date Event Stopped (DD/MM/YY)	Event Description
___/___/___	___/___/___	
___/___/___	___/___/___	
___/___/___	___/___/___	
___/___/___	___/___/___	

L. Referrals & Comments

Date (DD/MM/YY)	Referral	Comment (if referred, where and what for)
___/___/___	☐Yes ☐No	
___/___/___	☐Yes ☐No	
___/___/___	☐Yes ☐No	
___/___/___	☐Yes ☐No	
___/___/___	☐Yes ☐No	
___/___/___	☐Yes ☐No	
___/___/___	☐Yes ☐No	
___/___/___	☐Yes ☐No	

J. Patient Status

☐Active ☐Dead (Date: ___/___/___) ☐Migrated (Date: ___/___/___)

☐Unresponsive (Date: ___/___/___) ☐Inactive (Date: ___/___/___)

HTN Treatment Card

V1.2, 27 January 2020

A. Facility Information

Council Area:	Health Center ID:
Registration date (DD/MM/YY): ___/___/___	PHC Name / Ward:

B. Patient Identification Information

Program ID#: | Surname: | First Name:

Unique ID #: | 1st Phone Number:

Next of Kin Name: | Next of Kin Phone Number:

Sex: ☐Male ☐Female | Birth Date (DD/MM/YYYY): ___/___/_____ | Height: ___ cm

Education Level: ☐Never attended school ☐Primary School ☐Secondary School ☐High School ☐College/University ☐Professional Degree ☐Other: ____________

C. Diagnosis & Co-morbidity

Event/Condition	At registration: Yes	No	Unknown	Date, if known (MM/YY)	During Program: New Event	Date, if known (DD/MM/YY)
Hypertension diagnosis	☐	☐	NA	___/___	NA	NA
Diabetes mellitus diagnosis	☐	☐	☐	___/___	☐	___/___/___
Heart failure diagnosis	☐	☐	☐	___/___	☐	___/___/___
Chronic kidney disease diagnosis	☐	☐	☐	___/___	☐	___/___/___
Prior stroke	☐	☐	☐	___/___	☐	___/___/___
Prior heart attack	☐	☐	☐	___/___	☐	___/___/___
Angiotensin Receptor Blocker Allergy	☐	☐	☐	___/___	☐	___/___/___
History of smoking/tobacco	☐	☐	☐	☐Current<br>☐Former	☐Less than 1x per week<br>☐Less than 1x per day<br>☐More than 1x per day	
History of alcohol use	☐	☐	☐	☐Current<br>☐Former	☐Less than 1 bottle per week<br>☐Between 1 bottle per week and 1 bottle per day<br>☐More than 1 unit per day	

D. Pregnancy, complete among women only for each new pregnancy including current pregnancy at registration

Pregnant at registration: ☐Yes ☐No	Date of delivery or cessation: ___/___/_____
Visit date with new pregnancy: ___/___/_____	Date of delivery or cessation: ___/___/_____
Visit date with new pregnancy: ___/___/_____	Date of delivery or cessation: ___/___/_____

E. Hypertensive medications taken at baseline, complete one line per medication

How long has the drug been taken?	Drug	Dose	Other Drug	Stop Date (DD/MM/YY)
☐Less than 1 year<br>☐1 to 5 years<br>☐More than 5 years	☐Amlodipine<br>☐Losartan<br>☐Hydrochlorothiazide	☐5 mg ☐50 mg<br>☐10 mg ☐100 mg<br>☐25 mg	Name: ________<br>Dose: ________	___/___/___
☐Less than 1 year<br>☐1 to 5 years<br>☐More than 5 years	☐Amlodipine<br>☐Losartan<br>☐Hydrochlorothiazide	☐5 mg ☐50 mg<br>☐10 mg ☐100 mg<br>☐25 mg	Name: ________<br>Dose: ________	___/___/___
☐Less than 1 year<br>☐1 to 5 years<br>☐More than 5 years	☐Amlodipine<br>☐Losartan<br>☐Hydrochlorothiazide	☐5 mg ☐50 mg<br>☐10 mg ☐100 mg<br>☐25 mg	Name: ________<br>Dose: ________	___/___/___
☐Less than 1 year<br>☐1 to 5 years<br>☐More than 5 years	☐Amlodipine<br>☐Losartan<br>☐Hydrochlorothiazide	☐5 mg ☐50 mg<br>☐10 mg ☐100 mg<br>☐25 mg	Name: ________<br>Dose: ________	___/___/___

F. Blood Pressure Measures, complete 2 blood pressure measures at each visit including registration

Visit Date (DD/MM/YY)	Weight, kg	SBP, mmHg	DBP, mmHg	SBP, mmHg	DBP, mmHg	Heart rate, bpm	Med Change	Side Effect	Next Visit Date (DD/MM/YY)
__ __/__ __/__ __	__ __ __	__ __ __	__ __ __	__ __ __	__ __ __	__ __ __	☐Yes ☐No	☐Yes ☐No	__ __/__ __/__ __
__ __/__ __/__ __	__ __ __	__ __ __	__ __ __	__ __ __	__ __ __	__ __ __	☐Yes ☐No	☐Yes ☐No	__ __/__ __/__ __
__ __/__ __/__ __	__ __ __	__ __ __	__ __ __	__ __ __	__ __ __	__ __ __	☐Yes ☐No	☐Yes ☐No	__ __/__ __/__ __
__ __/__ __/__ __	__ __ __	__ __ __	__ __ __	__ __ __	__ __ __	__ __ __	☐Yes ☐No	☐Yes ☐No	__ __/__ __/__ __
__ __/__ __/__ __	__ __ __	__ __ __	__ __ __	__ __ __	__ __ __	__ __ __	☐Yes ☐No	☐Yes ☐No	__ __/__ __/__ __
__ __/__ __/__ __	__ __ __	__ __ __	__ __ __	__ __ __	__ __ __	__ __ __	☐Yes ☐No	☐Yes ☐No	__ __/__ __/__ __
__ __/__ __/__ __	__ __ __	__ __ __	__ __ __	__ __ __	__ __ __	__ __ __	☐Yes ☐No	☐Yes ☐No	__ __/__ __/__ __
__ __/__ __/__ __	__ __ __	__ __ __	__ __ __	__ __ __	__ __ __	__ __ __	☐Yes ☐No	☐Yes ☐No	__ __/__ __/__ __
__ __/__ __/__ __	__ __ __	__ __ __	__ __ __	__ __ __	__ __ __	__ __ __	☐Yes ☐No	☐Yes ☐No	__ __/__ __/__ __
__ __/__ __/__ __	__ __ __	__ __ __	__ __ __	__ __ __	__ __ __	__ __ __	☐Yes ☐No	☐Yes ☐No	__ __/__ __/__ __
__ __/__ __/__ __	__ __ __	__ __ __	__ __ __	__ __ __	__ __ __	__ __ __	☐Yes ☐No	☐Yes ☐No	__ __/__ __/__ __
__ __/__ __/__ __	__ __ __	__ __ __	__ __ __	__ __ __	__ __ __	__ __ __	☐Yes ☐No	☐Yes ☐No	__ __/__ __/__ __
__ __/__ __/__ __	__ __ __	__ __ __	__ __ __	__ __ __	__ __ __	__ __ __	☐Yes ☐No	☐Yes ☐No	__ __/__ __/__ __
__ __/__ __/__ __	__ __ __	__ __ __	__ __ __	__ __ __	__ __ __	__ __ __	☐Yes ☐No	☐Yes ☐No	__ __/__ __/__ __
__ __/__ __/__ __	__ __ __	__ __ __	__ __ __	__ __ __	__ __ __	__ __ __	☐Yes ☐No	☐Yes ☐No	__ __/__ __/__ __
__ __/__ __/__ __	__ __ __	__ __ __	__ __ __	__ __ __	__ __ __	__ __ __	☐Yes ☐No	☐Yes ☐No	__ __/__ __/__ __
__ __/__ __/__ __	__ __ __	__ __ __	__ __ __	__ __ __	__ __ __	__ __ __	☐Yes ☐No	☐Yes ☐No	__ __/__ __/__ __
__ __/__ __/__ __	__ __ __	__ __ __	__ __ __	__ __ __	__ __ __	__ __ __	☐Yes ☐No	☐Yes ☐No	__ __/__ __/__ __
__ __/__ __/__ __	__ __ __	__ __ __	__ __ __	__ __ __	__ __ __	__ __ __	☐Yes ☐No	☐Yes ☐No	__ __/__ __/__ __
__ __/__ __/__ __	__ __ __	__ __ __	__ __ __	__ __ __	__ __ __	__ __ __	☐Yes ☐No	☐Yes ☐No	__ __/__ __/__ __
__ __/__ __/__ __	__ __ __	__ __ __	__ __ __	__ __ __	__ __ __	__ __ __	☐Yes ☐No	☐Yes ☐No	__ __/__ __/__ __
__ __/__ __/__ __	__ __ __	__ __ __	__ __ __	__ __ __	__ __ __	__ __ __	☐Yes ☐No	☐Yes ☐No	__ __/__ __/__ __
__ __/__ __/__ __	__ __ __	__ __ __	__ __ __	__ __ __	__ __ __	__ __ __	☐Yes ☐No	☐Yes ☐No	__ __/__ __/__ __
__ __/__ __/__ __	__ __ __	__ __ __	__ __ __	__ __ __	__ __ __	__ __ __	☐Yes ☐No	☐Yes ☐No	__ __/__ __/__ __
__ __/__ __/__ __	__ __ __	__ __ __	__ __ __	__ __ __	__ __ __	__ __ __	☐Yes ☐No	☐Yes ☐No	__ __/__ __/__ __
__ __/__ __/__ __	__ __ __	__ __ __	__ __ __	__ __ __	__ __ __	__ __ __	☐Yes ☐No	☐Yes ☐No	__ __/__ __/__ __
__ __/__ __/__ __	__ __ __	__ __ __	__ __ __	__ __ __	__ __ __	__ __ __	☐Yes ☐No	☐Yes ☐No	__ __/__ __/__ __
__ __/__ __/__ __	__ __ __	__ __ __	__ __ __	__ __ __	__ __ __	__ __ __	☐Yes ☐No	☐Yes ☐No	__ __/__ __/__ __
__ __/__ __/__ __	__ __ __	__ __ __	__ __ __	__ __ __	__ __ __	__ __ __	☐Yes ☐No	☐Yes ☐No	__ __/__ __/__ __
__ __/__ __/__ __	__ __ __	__ __ __	__ __ __	__ __ __	__ __ __	__ __ __	☐Yes ☐No	☐Yes ☐No	__ __/__ __/__ __
__ __/__ __/__ __	__ __ __	__ __ __	__ __ __	__ __ __	__ __ __	__ __ __	☐Yes ☐No	☐Yes ☐No	__ __/__ __/__ __
__ __/__ __/__ __	__ __ __	__ __ __	__ __ __	__ __ __	__ __ __	__ __ __	☐Yes ☐No	☐Yes ☐No	__ __/__ __/__ __
__ __/__ __/__ __	__ __ __	__ __ __	__ __ __	__ __ __	__ __ __	__ __ __	☐Yes ☐No	☐Yes ☐No	__ __/__ __/__ __
__ __/__ __/__ __	__ __ __	__ __ __	__ __ __	__ __ __	__ __ __	__ __ __	☐Yes ☐No	☐Yes ☐No	__ __/__ __/__ __
__ __/__ __/__ __	__ __ __	__ __ __	__ __ __	__ __ __	__ __ __	__ __ __	☐Yes ☐No	☐Yes ☐No	__ __/__ __/__ __
__ __/__ __/__ __	__ __ __	__ __ __	__ __ __	__ __ __	__ __ __	__ __ __	☐Yes ☐No	☐Yes ☐No	__ __/__ __/__ __
__ __/__ __/__ __	__ __ __	__ __ __	__ __ __	__ __ __	__ __ __	__ __ __	☐Yes ☐No	☐Yes ☐No	__ __/__ __/__ __
__ __/__ __/__ __	__ __ __	__ __ __	__ __ __	__ __ __	__ __ __	__ __ __	☐Yes ☐No	☐Yes ☐No	__ __/__ __/__ __
__ __/__ __/__ __	__ __ __	__ __ __	__ __ __	__ __ __	__ __ __	__ __ __	☐Yes ☐No	☐Yes ☐No	__ __/__ __/__ __
__ __/__ __/__ __	__ __ __	__ __ __	__ __ __	__ __ __	__ __ __	__ __ __	☐Yes ☐No	☐Yes ☐No	__ __/__ __/__ __
__ __/__ __/__ __	__ __ __	__ __ __	__ __ __	__ __ __	__ __ __	__ __ __	☐Yes ☐No	☐Yes ☐No	__ __/__ __/__ __
__ __/__ __/__ __	__ __ __	__ __ __	__ __ __	__ __ __	__ __ __	__ __ __	☐Yes ☐No	☐Yes ☐No	__ __/__ __/__ __
__ __/__ __/__ __	__ __ __	__ __ __	__ __ __	__ __ __	__ __ __	__ __ __	☐Yes ☐No	☐Yes ☐No	__ __/__ __/__ __
__ __/__ __/__ __	__ __ __	__ __ __	__ __ __	__ __ __	__ __ __	__ __ __	☐Yes ☐No	☐Yes ☐No	__ __/__ __/__ __
__ __/__ __/__ __	__ __ __	__ __ __	__ __ __	__ __ __	__ __ __	__ __ __	☐Yes ☐No	☐Yes ☐No	__ __/__ __/__ __
__ __/__ __/__ __	__ __ __	__ __ __	__ __ __	__ __ __	__ __ __	__ __ __	☐Yes ☐No	☐Yes ☐No	__ __/__ __/__ __
__ __/__ __/__ __	__ __ __	__ __ __	__ __ __	__ __ __	__ __ __	__ __ __	☐Yes ☐No	☐Yes ☐No	__ __/__ __/__ __
__ __/__ __/__ __	__ __ __	__ __ __	__ __ __	__ __ __	__ __ __	__ __ __	☐Yes ☐No	☐Yes ☐No	__ __/__ __/__ __
__ __/__ __/__ __	__ __ __	__ __ __	__ __ __	__ __ __	__ __ __	__ __ __	☐Yes ☐No	☐Yes ☐No	__ __/__ __/__ __

G. Hypertensive medications prescribed, complete one line per medication, when medications are changed, update this section.

Date Prescribed (DD/MM/YY)	Amlodipine	Losartan	Hydrochlo-rothiazide	Other	Reason Stopped	Stop Date (DD/MM/YY)
__ __/__ __/__ __	☐5 mg ☐10 mg	☐50 mg ☐100 mg	☐12.5 mg ☐25 mg	Name:__________ Dose:__________	☐Contraindicated ☐Side Effects ☐Dose Changed ☐Other: __________	__ __/__ __/__ __
__ __/__ __/__ __	☐5 mg ☐10 mg	☐50 mg ☐100 mg	☐12.5 mg ☐25 mg	Name:__________ Dose:__________	☐Contraindicated ☐Side Effects ☐Dose Changed ☐Other: __________	__ __/__ __/__ __
__ __/__ __/__ __	☐5 mg ☐10 mg	☐50 mg ☐100 mg	☐12.5 mg ☐25 mg	Name:__________ Dose:__________	☐Contraindicated ☐Side Effects ☐Dose Changed ☐Other: __________	__ __/__ __/__ __
__ __/__ __/__ __	☐5 mg ☐10 mg	☐50 mg ☐100 mg	☐12.5 mg ☐25 mg	Name:__________ Dose:__________	☐Contraindicated ☐Side Effects ☐Dose Changed ☐Other: __________	__ __/__ __/__ __
__ __/__ __/__ __	☐5 mg ☐10 mg	☐50 mg ☐100 mg	☐12.5 mg ☐25 mg	Name:__________ Dose:__________	☐Contraindicated ☐Side Effects ☐Dose Changed ☐Other: __________	__ __/__ __/__ __
__ __/__ __/__ __	☐5 mg ☐10 mg	☐50 mg ☐100 mg	☐12.5 mg ☐25 mg	Name:__________ Dose:__________	☐Contraindicated ☐Side Effects ☐Dose Changed ☐Other: __________	__ __/__ __/__ __

H. Counseling, complete each time counseling is provided

Date of Counseling (DD/MM/YY)	Diet	Activity	Alcohol	Tobacco	Medication Adherence	Other
__ __/__ __/__ __	☐ Yes ☐ No	☐ Yes ☐ No	☐ Yes ☐ No	☐ Yes ☐ No	☐ Yes ☐ No	________________
__ __/__ __/__ __	☐ Yes ☐ No	☐ Yes ☐ No	☐ Yes ☐ No	☐ Yes ☐ No	☐ Yes ☐ No	________________
__ __/__ __/__ __	☐ Yes ☐ No	☐ Yes ☐ No	☐ Yes ☐ No	☐ Yes ☐ No	☐ Yes ☐ No	________________
__ __/__ __/__ __	☐ Yes ☐ No	☐ Yes ☐ No	☐ Yes ☐ No	☐ Yes ☐ No	☐ Yes ☐ No	________________
__ __/__ __/__ __	☐ Yes ☐ No	☐ Yes ☐ No	☐ Yes ☐ No	☐ Yes ☐ No	☐ Yes ☐ No	________________
__ __/__ __/__ __	☐ Yes ☐ No	☐ Yes ☐ No	☐ Yes ☐ No	☐ Yes ☐ No	☐ Yes ☐ No	________________
__ __/__ __/__ __	☐ Yes ☐ No	☐ Yes ☐ No	☐ Yes ☐ No	☐ Yes ☐ No	☐ Yes ☐ No	________________
__ __/__ __/__ __	☐ Yes ☐ No	☐ Yes ☐ No	☐ Yes ☐ No	☐ Yes ☐ No	☐ Yes ☐ No	________________
__ __/__ __/__ __	☐ Yes ☐ No	☐ Yes ☐ No	☐ Yes ☐ No	☐ Yes ☐ No	☐ Yes ☐ No	________________
__ __/__ __/__ __	☐ Yes ☐ No	☐ Yes ☐ No	☐ Yes ☐ No	☐ Yes ☐ No	☐ Yes ☐ No	________________
__ __/__ __/__ __	☐ Yes ☐ No	☐ Yes ☐ No	☐ Yes ☐ No	☐ Yes ☐ No	☐ Yes ☐ No	________________
__ __/__ __/__ __	☐ Yes ☐ No	☐ Yes ☐ No	☐ Yes ☐ No	☐ Yes ☐ No	☐ Yes ☐ No	________________
__ __/__ __/__ __	☐ Yes ☐ No	☐ Yes ☐ No	☐ Yes ☐ No	☐ Yes ☐ No	☐ Yes ☐ No	________________
__ __/__ __/__ __	☐ Yes ☐ No	☐ Yes ☐ No	☐ Yes ☐ No	☐ Yes ☐ No	☐ Yes ☐ No	________________

I. Lab Data, complete each time new lab data are available

Results Date (DD/MM/YY)	Serum creati-nine, mg/dL	Serum potassi-um, mEq/L	Serum sodi-um, mEq/L	Total cholesterol, mg/dL	Spot microalbu-min, mg	Hemoglobin A1C, %
__ __/__ __/__ __	__ __ . __ ☐µmol/l	__ __ . __ ☐mmol/l	__ __ __ ☐mmol/l	__ __ __ ☐mmol/l	__ __ __	__ __ . __
__ __/__ __/__ __	__ __ . __ ☐µmol/l	__ __ . __ ☐mmol/l	__ __ __ ☐mmol/l	__ __ __ ☐mmol/l	__ __ __	__ __ . __
__ __/__ __/__ __	__ __ . __ ☐µmol/l	__ __ . __ ☐mmol/l	__ __ __ ☐mmol/l	__ __ __ ☐mmol/l	__ __ __	__ __ . __
__ __/__ __/__ __	__ __ . __ ☐µmol/l	__ __ . __ ☐mmol/l	__ __ __ ☐mmol/l	__ __ __ ☐mmol/l	__ __ __	__ __ . __
__ __/__ __/__ __	__ __ . __ ☐µmol/l	__ __ . __ ☐mmol/l	__ __ __ ☐mmol/l	__ __ __ ☐mmol/l	__ __ __	__ __ . __
__ __/__ __/__ __	__ __ . __ ☐µmol/l	__ __ . __ ☐mmol/l	__ __ __ ☐mmol/l	__ __ __ ☐mmol/l	__ __ __	__ __ . __
__ __/__ __/__ __	__ __ . __ ☐µmol/l	__ __ . __ ☐mmol/l	__ __ __ ☐mmol/l	__ __ __ ☐mmol/l	__ __ __	__ __ . __
__ __/__ __/__ __	__ __ . __ ☐µmol/l	__ __ . __ ☐mmol/l	__ __ __ ☐mmol/l	__ __ __ ☐mmol/l	__ __ __	__ __ . __
__ __/__ __/__ __	__ __ . __ ☐µmol/l	__ __ . __ ☐mmol/l	__ __ __ ☐mmol/l	__ __ __ ☐mmol/l	__ __ __	__ __ . __
