# Supplementary material for: Hypertension Treatment in Nigeria (HTN) Program: rationale and design for a type 2 hybrid, effectiveness, and implementation interrupted time series trial
Source: Implement Sci Commun. 2022 Aug 2;3:84. doi: 10.1186/s43058-022-00328-9 (PMC9344662; doi:10.1186/s43058-022-00328-9)
Supplement: Supplementary file 5 — Additional file 5. HTN Program Training. Detailed listing of training provided to HTN Program community health extension workers, community health officers, record officers, pharmacists, and other healthcare workers. [file 43058_2022_328_MOESM5_ESM.docx]

**Additional Table 1.** Training for the Hypertension Treatment in Nigeria Program

| **Training** | **Participants** | **Duration** | **Location** | **Facilitator** | **Evaluation** |
| --- | --- | --- | --- | --- | --- |
| **CHEWs and non-physician health workers** | | | | | |
| Initial HTN sensitization: introduction to the HTN Program | ≥2-CHEWs per PHC | 2-days | UATH | PI or Co-I | Pre- and post-tests for knowledge of appropriate pressure measure, treatment, and patient management |
| Refresher training on the HTN Program, BP measurement, and hypertension treatment | ≥2-CHEWs per PHC | 2-days | UATH | PI or Co-I | None |
| Refresher training on the use of patient treatment card to capture patient information | ≥ 2 non-physician health-workers per PHC | ½ day per session staggered over a six-day period | UATH | Co-I and Research Team | Mock data entry |
| Targeted refresher training based on data quality and emergent issues from supportive supervision visits | ≥4 non-physician health workers from each PHC | ½ day per session staggered over a seven-day period | UATH | Co-I and Research Team | None |
| Medication prescription and medication management | ≥4 non-physician health workers per PHC | ½ day per session staggered over a three-day period | UATH | Co-I and Research Team | Mock medication data entry |
| Refresher training on medication prescription and medication management | ≥ 2 non-physician health workers per PHC | ½ day per session staggered over a three-day period | UATH | Co-I and Research Team | Mock medication data entry |
| Home BP monitoring and health coaching | 3 CHEWs and 1 facility managers per PHC, from 10 selected PHCs | 1-day | UATH | Co-I and Research Team | Pre- and post-tests for knowledge of appropriate pressure measure, motivational interviewing, and home visit procedures |
| Refresher training on home BP monitoring and health coaching | CHEWs and 1 facility managers per PHC, from 10 selected PHCs | 1-day | UATH | Co-I and Research Team | None |
| Initial HTN sensitization: introduction to the HTN Program | ≥2 non-physician health workers from each PHC who are not participating in the study | 1-day session staggered over a six-day period | UATH | Co-I and Research Team | Pre- and post-tests for knowledge of appropriate pressure measure, treatment, and patient management |
| **Record Officers** | | | | | |
| Data entry and electronic data capture | ≥1 record officer per PHC | ½ day | UATH | Co-I and Research Team | Mock data entry |
| Refresher training on data entry and electronic data capture | ≥1 record officer per PHC | ½ day per session staggered over a ten-day period | UATH | Co-I and Research Team | Mock data entry |
| Data query resolution | ≥1 record officer per PHC | ½ day per session staggered over a four-day period | UATH | Co-I and Research Team | Mock data entry |
| **Site Supervisors** | | | | | |
| Site monitoring | ≥3 site supervisors per area council | ½ day | UATH | Co-I and Research Team | None |
| **Pharmacists** | | | | | |
| Medication management and the HTN Program treatment protocol | 1 pharmacist each per local government council | ½ day | UATH | Co-I and Research Team | None |
| Medication management and the HTN Program treatment protocol | ≥1 pharmacist or pharmacy technician per area council | ½ day | UATH | Co-I and Research Team | Mock medication data entry |
| Refresher training on medication management and the HTN Program treatment protocol | ≥ 1 pharmacy focal person and 1 facility manager per PHC | ½ day | UATH | Co-I and Research Team | Mock medication data entry |
| Drug Revolving Fund | ≥1 pharmacy technician per PHC | 2-days | UATH | Pharmacist and Co-Is | Practical demonstration of understanding of documents and documentation of medication data |
| **Health Educators** | | | | | |
| Community mobilization training and ongoing quarterly meetings | ≥1 health educator in each government council | ½ day, and held quarterly | UATH | Co-I | None |
| **Laboratory Scientist** | | | | | |
| Collection, management, and data entry for biospecimens | ≥1 laboratory scientist from 10 selected PHCs | ½ day | UATH | Co-I | None |

Abbreviations: CHEW, Community Health Extension Worker; Co-I, Co-investigator; PHC, Primary Health Care Center; PI, Principal Investigator; UATH, University of Abuja Teaching Hospital
