# Supplementary material for: Hypertension Treatment in Nigeria (HTN) Program: rationale and design for a type 2 hybrid, effectiveness, and implementation interrupted time series trial
Source: Implement Sci Commun. 2022 Aug 2;3:84. doi: 10.1186/s43058-022-00328-9 (PMC9344662; doi:10.1186/s43058-022-00328-9)
Supplement: Supplementary file 6 — Additional file 6. HTN Program Statistical Analysis Plan. Statistical analysis plan for the HTN Program. [file 43058_2022_328_MOESM6_ESM.pdf]

STATISTICAL ANALYSIS PLAN (SAP)

HTN:
Hypertension Treatment in Nigeria

May 23, 2022

Version 2.0

IRB Number: FHREC/2019/01/03/16-01-19

List of Abbreviations

AE	Adverse Event
CHEW	Community Health Extension Workers
CRF	Case Report Form
DMP	Data Management Plan
DSMB	Data and Safety Monitoring Board
DSMP	Data and Safety Monitoring Plan
DSQR	Data Status and Quality Reports
eCRF	Electronic Case Report Form
MOP	Manual of Operating Procedures
REDCap	Research Electronic Data Capture
SAE	Serious Adverse Event

STATISTICAL ANALYSIS PLAN (SAP)

HTN:
Hypertension Treatment in Nigeria

Co-Principal Investigator: Mark D. Huffman, MD, MPH
Co-Principal Investigator: Dike Ojji, MD, PhD

1. INTRODUCTION

The purpose of this document is to outline the proposed analyses for the Hypertension Treatment in Nigeria (HTN) Program. The HTN Program is evaluated through a type 2 hybrid, interrupted time series design with the aim of assessing effectiveness and implementation of a large-scale, system-level hypertension treatment and control program in Federal Capital Territory, Nigeria. The program is sponsored by the NIH / NHLBI through grant award #R01HL144708; Co-Principal Investigators: Mark D. Huffman, MD, MPH (contact PI) and Dike Ojji, MD, PhD. The principal investigators and coordinating activities for the program are located at the University of Abuja Teaching Hospital in Abuja, Nigeria and Northwestern University in the Department of Preventive Medicine, Feinberg School of Medicine in Chicago, IL, USA. The program activities will occur at selected primary health care centers within the Federal Capital Territory, Nigeria. The HTN Operations Team, comprised of leadership at Northwestern University and University of Abuja Teaching Hospital, oversees the general scientific direction of the program, including implementation and conduct.

<u>Study Aims</u>
The overarching study aims are as follows:

Formative Aim 1: Develop implementation pathways and intervention packages for a system-level hypertension program adapted from Kaiser Permanente's Northern California (KPNC) Hypertension Program and the World Health Organization (WHO) HEARTS package as exemplars for large-scale hypertension control for use in public, primary health care facilities in Federal Capital Territory [Abuja], Nigeria.

Aim 2: Evaluate the effectiveness of a system-level hypertension program on system-level hypertension treatment and control rates in the Federal Capital Territory [Abuja], Nigeria.

We hypothesize that this intervention will improve system- and patient-level treatment and control rates.

Aim 3: Evaluate the reach, adoption, implementation, maintenance, acceptability, and cost of the system-level hypertension program.

We hypothesize that this intervention will reach the target population and be adopted, implemented, maintained, acceptable, and affordable at the system- and patient-levels.

This SAP will focus on the details of analyses for Aims 2 and 3.

Study time points include the baseline control phase (months 1 through 9) and the intervention phase (months 10 through 48).

2. STUDY OUTCOMES

For all outcomes, participation is defined by site engagement in training, completed site initiation, and enrollment of at least 1 patient during the program.

<u>Primary Effectiveness Outcomes</u>
The co-primary effectiveness outcomes include:

- Change in slope from baseline slope of monthly hypertension treatment rates among participating primary health centers using any BP lowering drug.
- Change in slope from baseline slope of monthly hypertension control (defined as SBP <140 mmHg and DBP <90 mmHg) rates among participating primary health centers.

<u>Primary Implementation Outcomes</u>
The co-primary implementation outcomes include:

- Reach
- Effectiveness (2° effectiveness outcome)
- Adoption
- Implementation
- Maintenance
- Acceptability
- Cost of the system-level hypertension program

Table. Implementation outcomes for HTN Program.

RE-AIM Domain: Definition	Level	Type	Outcome
Reach: Absolute number, proportion, and representativeness of sites and individuals who participate in the HTN program	Program	Quantitative	• Number of participating PHCs/total number of selected PHCs in the FCT
	Center	Quantitative	• Diversity of participating PHCs and staff in terms of size, ward, baseline staffing levels
		Qualitative	• Reasons for non-participation of selected PHCs in the FCT<br>• Reasons for adult patients to have not been screened for high BP within participating PHCs within the past 3 working days
	Individual	Quantitative	• Number of adult patients with BPs measured / total number of adult patients within participating PHCs within the past 3 working days<br>• Differences in sociodemographic (e.g. age, sex, geography) characteristics between registered patients and individuals in the clinic catchment areas based on concurrently collected or community-based survey data<br>• Diversity of registered patients receiving care at participating PHCs for HTN diagnosis and management by age, sex, ward, and education
Effectiveness: The impact of the HTN program on treatment and control rates	Program	Quantitative	• Treatment rate within the overall system of participating PHCs defined by 6-month rolling average<br>• Control rate within the overall system of participating PHCs defined by 6-month rolling average<br>• Mean SBP and DBP within the overall system of participating PHCs defined by 6-month rolling average and based on last visit

	Center	Quantitative	• Median and/or mean treatment rate across participating PHCs defined by 6-month rolling average<br>• Median and/or mean control rate across participating PHCs defined by 6-month rolling average<br>• Mean SBP and DBP across participating PHCs defined by 6-month rolling average and based on last visit
		Qualitative	• Reasons for variation in treatment rates between participating PHCs<br>• Reasons for variation in control rates between participating PHCs<br>• Reasons for variation in mean systolic and diastolic blood pressure between participating PHCs
Adoption: Absolute number, proportion, and representativeness of sites who are willing to initiate the HTN program	Program	Quantitative	• Percentage of PHCs using the Hypertension Patient Registry in the last 3-months<br>• Percentage of patients treated with fixed dose combination therapies in the last 3-months
		Qualitative	• Reasons for variation in registry use among participating PHCs at 3-months after site initiation<br>• Reasons for variation in use of fixed dose combination therapies in the last 3-months<br>• Adoption of team based care among participating PHCs, and reasons for success or challenges
Implementation: Fidelity to the HTN program protocol, including consistency of delivery as intended. Time and cost of the intervention, and use of the intervention strategies	Program	Quantitative	Fidelity (Implementation)<br>• Proportion of selected PHCs who participated in baseline hypertension training<br>• Proportion of selected PHCs who participated in site initiation training<br>• Proportion of selected PHCs who received at least one supportive supervision visit in the past 7-months<br>• Proportion of selected PHCs who received an audit and feedback report within the past 3-months<br>• Percentage of PHCs with a working blood pressure monitor at the site on the day of assessment<br>• Percentage of PHCs with blood pressure medicines available on the day of assessment<br>• Percentage of patients with step up indicated who received step up treatment in the last 6-months<br>Cost<br>• Modeled direct HTN program costs based on staff, BP machines, data capture, data analysis, and BP lowering drugs for hypertension diagnosis, treatment and control overall, for each PHC and per patient
	Program	Qualitative	Fidelity (Implementation)<br>• Reasons for variation in fidelity measures<br>• Reasons for variation in availability of essentials medicines and equipment<br>• Reasons for variation in fidelity to the step up treatment protocol<br>Cost<br>• Acceptability of upfront and ongoing HTN program costs among stakeholders, including within Federal Ministry of Health
	Center	Quantitative	Fidelity (Intervention)

			• Number and proportion of adult patients with hypertension who are registered/total number of adult patients with elevated blood pressure within participating PHCs within the past 3 working days<br>• Monthly proportion of registered patients with appropriate stepped care/total number of registered patients<br>• Monthly proportion of registered patients treated with fixed dose combination therapy/total number of patients on treatment
	Center, Individual	Qualitative	Fidelity (Implementation)<br>• Reasons for adult patients with hypertension to have not been registered within participating PHCs within the past 3 working days
	Individual	Quantitative	Cost<br>• Modeled monthly and annual out-of-pocket drug costs for hypertension treatment
	Individual	Qualitative	Acceptability<br>• Reasons for variation in acceptability, satisfaction, and perceived quality of care at patient-level<br>• Trust in primary health care system<br>Cost<br>• Acceptability of upfront and ongoing HTN diagnosis and treatment costs among patients with HTN
Maintenance: The extent to which the HTN program becomes institutionalized or part of the routine organizational practice	Center	Quantitative	Maintenance<br>• Proportion of participating PHCs who maintain treatment rates above baseline rates at 6, 12, 24, 36, and 48 months<br>• Proportion of participating PHCs who maintain control rates above baseline rates at 6, 12, 24, 36, and 48 months<br>• Proportion of participating PHCs without blood pressure medication stockouts at 36 and 48 months<br>• Proportion of participants retained in care at participating PHCs at 6, 12, 24, 36, and 48 months
		Qualitative	Maintenance<br>• Reasons for variation in maintenance of treatment rates above baseline rates<br>• Reasons for variation in maintenance of control rates above baseline rates<br>• Reasons for variation in sustainment of blood pressure medication supplies<br>• Reasons for variation in proportion of participants retained in care at PHCs
	Individual	Qualitative	Maintenance<br>• Reasons for remaining in care and on treatment within the PHC

<u>Secondary Outcomes</u>

Secondary effectiveness outcomes include:

- Mean SBP and DBP among eligible clinic patients with hypertension
- Rates of single versus double or triple blood pressure lowering medication use (including fixed-dose combination use)

<u>Safety Outcomes</u>

Safety outcomes include:

- Proportion of participants with *any* potentially relevant side effect, including provider diagnosis of angioedema, acute kidney injury,[1] electrolyte abnormalities,[2] syncope, or dizziness
- Rate of relevant side effects at the participant level (i.e. count per participant)
- Proportion of participants with *any* SAE according to Good Clinical Practice (GCP) definition

[1]Defined as relative increase in serum creatinine by 50% or an absolute increase by 0.3 mg/dl (>0.26 umol/L)
[2]Potassium < 3.5 mEq/L or >5.5 mEq/L OR Sodium <125 mEq/L or > 145 mEq/L

3. DEMOGRAPHICS AND BASELINE ASSESSMENTS

The following are socio-demographic assessments of interest for analyses. To evaluate the effects of socio-demographics, we will use patient-level mixed effects models including random effects to account for clustering at the primary healthcare center level.

1) Age
2) Sex
3) Socioeconomic position defined by attained education level
4) Baseline history of hypertension
5) Baseline history of hypertension treatment
6) Baseline history of cardiovascular disease including hypertension, stroke, and heart attack
7) Primary health center ward

Note that some additional exploratory analyses may examine these additional demographic variables as covariates and/or effect modifiers as well. We will label any exploratory analyses involving additional potential covariates as *post hoc* in any dissemination materials.

4. DATA STORAGE

Data will be collected and managed using Research Electronic Data Capture (REDCap) housed at University of Abuja. REDCap is a secure, web-based application designed for research studies that provides an intuitive interface for validated data entry, audit trails for tracking data manipulation and export procedures, and automated export procedures for seamless data downloads to common statistical packages, and procedures for importing data from external sources. Refer to the study Data Management Plan (DMP) for details.

5. STATISTICAL METHODS

Descriptive statistics will summarize participant demographics and clinical outcomes (control and treatment rates) overall and by phase: proportion (percentages) for categorical variables; mean (± standard deviation) for continuous variables; and median (interquartile range) for skewed or count variables. Analyses in general will employ normal theory methods and residual diagnostics will evaluate validity of assumptions; where appropriate (i.e., in the event of low cell counts for categorical data or questions of normality), transformation of variables, nonparametric methods, or exact tests may be employed. All primary efficacy and safety analyses will be pre-specified as outlined in this SAP, and deviations from planned analyses or *post hoc* analyses will be labeled as such in any reports or dissemination materials.

Analyses will assume a two-sided 5% type I error rate unless otherwise specified; there will be some exploratory analyses that will involve a relaxed type I error rate (10%). There will be no corrections made for multiple hypothesis tests.

Planned Primary, Secondary, Safety Analyses

The co-primary effectiveness analyses are based on an interrupted time series design using segmented regression models, allowing a baseline control phase of 9 months (January 2020 to September 2020), and an intervention phase of approximately 39 months (October 2020 to December 2023). Each participating primary health center will be centered to time=0 when the intervention is implemented. The primary analysis will assume the intervention phase once on-site training has occurred for any component of the intervention. We will use Poisson regression models for both co-primary effectiveness outcome and adjust for confounders such as the composition of the study population (site characteristic, age group, gender). We will control for autocorrelation and seasonal changes data if exhibit seasonal fluctuations. Sensitivity analyses will consider the intervention phase as started once on-site training has occurred for the majority of intervention components, as well as assess for level changes in addition to slope change.

These analyses will assume the following for treatment (T) and control (C) at each month i (i=1…48) across all participating sites:

$$N_i = Number\ Newly\ Registered_i + Number\ Previously\ Registered\ Returned_i$$

The number of hypertensive registrants is defined as the number of patients with hypertension who are newly registered during any calendar month plus the total number of previously registered individuals who returned to primary health centers in that month for ongoing care. Registrants who return for follow up care more than one time in a calendar month will be counted once for this analysis.

$$T_i = \frac{Number\ Newly\ Prescribed_i + Number\ Continuing\ BP\ Lowering\ Medication_i}{N_i}$$

Treatment rates will be calculated each month across all participating sites. Treatment is defined at a patient level as an ongoing or new prescription of any BP-lowering medication during the calendar month.

$$C_i = \frac{Number\ with\ SBP < 140\ mmHg\ and\ DBP < 90\ mmHg_i}{N_i}$$

Control rates will be calculated each month across all participating sites. Control is defined at a patient level, as measured SBP <140 mmHg and DBP <90 mmHg during the calendar month.

Monthly sensitivity analyses will be performed to evaluate the proportion who achieve control of SBP <130 mmHg and DBP <80 mmHg based on 2017 US hypertension guidelines, which do not reflect the current standard of care in Nigeria. We will also evaluate more stringent definitions of hypertension control, including using a definition of control based on patients having 2 or more consecutive visits with SBP <140 mmHg and DBP <90 mmHg, as well as other WHO HEARTS outcomes, including retention in care, as defined in the Table.

To evaluate the individual effects of intervention components and time variance in implementation on our primary outcomes, we will use patient-level mixed effects models including random effects to account for clustering at the clinic level. We will perform sensitivity analyses by both excluding and restricting repeated measures of the same patients over the study period. We will use frailty models to evaluate the time to control of hypertension and associated factors. .

For implementation outcomes, we will use mixed methods analysis using the RE-AIM framework to triangulate both routinely collected quantitative data and qualitative data to evaluate the reach, effectiveness (based on secondary effectiveness outcomes) adoption, implementation, and maintenance, acceptability, and cost of the interventions.

Subgroup Analyses and Heterogeneity of Intervention Effects

Subgroup analyses will examine primary outcomes within and across sites based on staffing levels, staff training, geography, and drug availability.

Agreement between Outcomes

Sites with higher treatment rates are anticipated to also have higher control rates. We will assess agreement between these outcomes.

6. ANALYTIC DATASET

Analyses will include all sites with data during the control or intervention phases, regardless of adherence to the protocol. We will conduct a sensitivity analysis using a per protocol approach, which will evaluate control rates based on implementation of the intervention components.

Power and sample size considerations allowed for some missing data (20% of sites). We will examine rates of missing data for all variables and determine whether the rates vary by site or participant characteristics, etc. We will also examine the mechanism that lead to missing data. These summarizations and mechanism of attrition will inform potential biases resulting from missing data. Depending on mechanism of the missing data, we will use appropriate strategy for handling missing data. Mixed effects models planned for longitudinal analysis are generally robust for unbalanced data across study time points.

7. POWER AND SAMPLE SIZE CONSIDERATIONS

Using a conservatively high baseline hypertension treatment rate of 20% based on previous estimates in Nigeria, recruiting 1,200 participants per month across 50 sites over 9 months of baseline and 39 months of intervention provides over 80% power to detect a difference in slope of 0.57% per month compared to underlying trend of 0.10% per month, resulting in hypertension treatment rate of 42.5% at the end of 48 months. Similarly, using a conservative baseline hypertension control rate of 10%, we will have over 80% power to detect a difference in slope of 0.44% per month compared to underlying trend of 0.05% per month, resulting in hypertension control rate of 27.0% at the end of 48 months. These effect sizes are conservative based on the KPNC experience to account for differences in the program populations, baseline treatment and control rates, and sites. These baseline trends are also conservatively high so that we are not underpowered if the background hypertension control rates improve during the program period. We will have more power for larger effects, as well as most secondary/exploratory outcomes.

8. TECHNICAL DETAILS

The SAP is subject to version control, and we anticipate modifications to analytic plans be documented herein. As in any study, the analytic plan may change due to assumption violations, logistical issues, unexpected empirical distributions of study outcomes, or a combination thereof. In these cases, the SAP will be updated accordingly. All analyses will be performed via SAS version 9.4 or higher (The SAS Institute; Cary, NC) or R version 3.6.0 or higher (The R Foundation for Statistical Computing platform). Table and figure formatting and style may be dictated by mode of dissemination or specific target journal(s) for results dissemination.

9. TIMELINE FOR ANALYSES

The analysis plan does not include any formal interim statistical analyses involving hypothesis testing or any pre-specified stopping criteria for efficacy or futility on primary or secondary outcomes. Interim reports to the study team and Data and Safety Monitoring Board (DSMB) will consist of process measures such as protocol departures, intervention implementation, etc. and simple descriptive statistics on primary and safety outcomes of interest. In addition, weekly meetings with the study team will utilize central statistical monitoring techniques as a method of quality control and quality assurance for trial data on an ongoing basis. We foresee the DSMB requiring specific data listings or summarizations, but these will be specified at the time of the relevant DSMB meeting(s); at this time, however, we do not plan for formal statistical analyses involving hypothesis testing for DSMB interim review.

To preserve the integrity of the study, no formal statistical analyses will occur until the REDCap database has been locked and all queries/discrepancies resolved; the date of database lock will be documented.
