# Supplementary material for: Hypertension Treatment in Nigeria (HTN) Program: rationale and design for a type 2 hybrid, effectiveness, and implementation interrupted time series trial
Source: Implement Sci Commun. 2022 Aug 2;3:84. doi: 10.1186/s43058-022-00328-9 (PMC9344662; doi:10.1186/s43058-022-00328-9)
Supplement: Supplementary file 7 — Additional file 7. HTN Program Supervision Checklist. Checklist used by members of the HTN Program implementation team during usual supervision visits to participating sites. [file 43058_2022_328_MOESM7_ESM.pdf]

No.	Question	Result
Section 1: Facility Information		
001	Facility number	
001a	Date	Day __ __ Month __ __ Year __ __ __ __
001c	Date of Last Visit	Day __ __ Month __ __ Year __ __ __ __
001b	Monitor Name	______________________________
003	Name of facility	______________________________

Section 2: Staffing

No.	Count each staff member only once, on the basis of the highest technical or professional qualification.	A. How many staff are currently working in this facility?	B. Of the total number, how many are part-time in this facility?	C. How many staff have newly joined since the last visit?	D. How many staff have left since the last visit?	E. How many were trained today?
201	Generalist medical doctors					
202	Specialist medical doctors					
203	Non-physician clinicians / paramedical professionals					
204	Nursing professionals					
208	Pharmacists					
211	Laboratory technicians (medical and pathology)					
212	Community health extension workers					
213	Junior community health extension workers					
214	Community volunteers attached to the health facility					
215	Community health officer					
216	Other (Specify): ______________________					

Section 3: Patient Access and Capacity

Use site ledgers and the HTN registry to capture the following information. The time frame should be approximately 3 working days, inclusive of the start and end dates.

No.	Question	Result
329	Start Date	Day __ __ Month __ __ Year __ __ __ __
330	End Date	Day __ __ Month __ __ Year __ __ __ __
331	What is the total number of adult patient visits to the health facility within the given dates?	Total No. of Adult Patient Visits:
332	Of these adult patients, how many had their BP checked during their visit?	No. of Adult Patients with BP Checked:
333	Of the patients who had their BP checked, how many had high BP?	No. of Adult Patients with High BP:
334	Of the patients with high BP, how many were newly or already registered?	No. Newly or Already Registered:

Section 4h: Basic Equipment: All equipment must be available and functioning

No.	Question	Result						
	Please tell me if the following basic equipment and supplies used in the provision of client services are available and functional in this facility today.<br>Ask to see the items	A) Available<br>If observed or reported, complete section B			B) Functioning			
		Observed	Reported not seen	Not available	Yes	No	Don't know	
501	Adult weighing scale	1 ☐	2 ☐	3 ☐	1 ☐	2 ☐	8 ☐	
504	Measuring tape-height board / stadiometre	1 ☐	2 ☐	3 ☐	1 ☐	2 ☐	8 ☐	
507	Blood pressure apparatus (may be digital or manual sphygmomanometer with stethoscope)	1 ☐	2 ☐	3 ☐	1 ☐	2 ☐	8 ☐	
	Section 5: Available Services: All answers must be "Yes"							
2014	Are the registry and patient treatment cards for the HTN Program available in the facility today?	Yes 1 ☐<br>No 2 ☐						
2018	How many patient treatment cards were dispensed by the monitor to the facility today?	________Treatment Cards						
	Site Supervision							
	Section 12: Training							
7000	How many patient treatment cards were reviewed during the site monitoring visit today?	________Treatment Cards						
7001	Of the cards that were reviewed today, how many had errors that required revision?	________Treatment Cards						
7002	Were patient blood pressure measurements directly observed during the site visit today?	No 0 ☐<br>Yes 1 ☐						7005
7003	If patient blood pressure measurements were not observed, why not?	________________________________						
7004	If patient blood pressure measurements were not observed, was the staff asked to measure the blood pressure of the supervisor?	No 0 ☐<br>Yes 1 ☐						7007
7005	How many site staff were observed measuring blood pressure during the site visit today?	________Staff						
7006	Overall, were blood pressures measured per protocol?	No 0 ☐<br>Yes 1 ☐						
7007	Overall, was data captured per protocol?	No 0 ☐<br>Yes 1 ☐						
7008	Was retraining provided today on either blood pressure measurement or data capture?	No 0 ☐<br>Yes, blood pressure measurement 1 ☐<br>Yes, data capture 2 ☐<br>Yes, both 3 ☐						

Comments about the assessment:

No.	Question	Result

Signature of HTN team member: ______________________ Signature of site representative: ______________________

Section 7: Medicines and Commodities: Recorded for information only

If any of the following medications are in stock, record the quantity on hand today. If any have been dispensed in the last 30-days, including today, record the quantity of 30-day doses dispensed.

	Drug Class	Generic Name	Dosage (mg)	Number of 30-day doses in stock	Number of 30-day doses dispensed in last 30 days
4029	ACE Inhibitor	Lisinopril	10 mg	____________	______________
4030	ACE Inhibitor	Lisinopril	5 mg	____________	______________
4031	Diuretic	Hydrochlorothiazide	50 mg	____________	______________
4032	Diuretic	Hydrochlorothiazide	25 mg	____________	______________
4033	Diuretic	Hydrochlorothiazide	12.5 mg	____________	______________
4034	Diuretic	Spironolactone	50 mg	____________	______________
4035	Diuretic	Spironolactone	25 mg	____________	______________
4036	Diuretic	Spironolactone	12.5 mg	____________	______________
4037	Diuretic	Amiloride / Hydrochloride	5 mg / 50 mg	____________	______________
4038	CCB	Amlodipine	10 mg	____________	______________
4039	CCB	Amlodipine	5 mg	____________	______________
4040	CCB	Nifedipine	30 mg	____________	______________
4041	CCB	Nifedipine	20 mg	____________	______________
4042	Central Acting Agent	Methyldopa	250 mg	____________	______________
4043	Vasodilator	Hydralazine	20 mg	____________	______________
4044	Beta Blocker	Labetalol	200 mg	____________	______________
4045	Beta Blocker	Propranolol	40 mg	____________	______________
4046	Beta Blocker	Propranolol	10 mg	____________	______________
4047	ARB	Telmisartan	40 mg	____________	______________
4048	ARB	Telmisartan	80 mg	____________	______________
4049	ARB	Losartan	50 mg	____________	______________
4050	ARB/CCB	Losartan/Amlodipine	50 mg / 5mg	____________	______________
4051	ARB/CCB/Diuretic	Losartan/Amlodipine/ Hydrochlorothiazide	50 mg / 5mg/12.5 mg	____________	______________

Capture any other current HYPERTENSION medication inventory (including ACE inhibitors, Thiazides, Angiotensin Receptor Blockers[ARB], Other diuretics, Beta blockers, and Calcium channel blockers[CCB]), as well as fixed dose combinations not captured in the section above:

	Drug Class	Generic Name	Dosage (mg)	Number of 30-day doses in stock	Number of 30-day doses dispensed in last 30 days
4052	_____________________	_____________________	____________	______________	______________
4053	_____________________	_____________________	____________	______________	______________

No.	Question	Result			
4054	______________________	______________________	_____________	_______________	_______________
4055	______________________	______________________	_____________	_______________	_______________
4056	______________________	______________________	_____________	_______________	_______________
4057	______________________	______________________	_____________	_______________	_______________
4058	______________________	______________________	_____________	_______________	_______________
4059	______________________	______________________	_____________	_______________	_______________
4060	______________________	______________________	_____________	_______________	_______________
